# Alkaloids from Marine Invertebrates as Important Leads for Anticancer Drugs Discovery and Development

**DOI:** 10.3390/molecules191220391

**Published:** 2014-12-05

**Authors:** Concetta Imperatore, Anna Aiello, Filomena D’Aniello, Maria Senese, Marialuisa Menna

**Affiliations:** The NeaNat Group, Department of Pharmacy, University of Naples “Federico II”, Via D. Montesano 49, Napoli 80131, Italy; E-Mails: cimperat@unina.it (C.I.); aiello@unina.it (A.A.); filomena.daniello@unina.it (F.D.); maria.senese@unina.it (M.S.)

**Keywords:** alkaloids, marine invertebrates, anticancer, natural products, drug discovery

## Abstract

The present review describes research on novel natural antitumor alkaloids isolated from marine invertebrates. The structure, origin, and confirmed cytotoxic activity of more than 130 novel alkaloids belonging to several structural families (indoles, pyrroles, pyrazines, quinolines, and pyridoacridines), together with some of their synthetic analogs, are illustrated. Recent discoveries concerning the current state of the potential and/or development of some of them as new drugs, as well as the current knowledge regarding their modes of action, are also summarized. A special emphasis is given to the role of marine invertebrate alkaloids as an important source of leads for anticancer drug discovery.

## 1. Introduction

Oceans cover more than 70% of the planet and provide a wealth of organisms that produce structurally unique bioactive metabolites. These molecules are originated from the phenomenon of biodiversity, in which the interactions among species and with the environment are devolved upon diverse complex chemical entities within the organisms that enhance their survival and competitiveness. The therapeutic area of infectious diseases and oncology have benefited greatly from this chemical arsenal and, as with plants, researchers have recognized its potential use to kill bacteria or cancer cells. Incidence of biological activity in compounds derived from marine sources is high, especially with regard to cytotoxicity, where extracts of marine species surpass those of terrestrial origin. Marine natural products are able to interact with many specific targets within the cell and are finding increasing use as probes to interrogate biological systems as part of chemical genomics and related research. Most of the bioactive molecules from the sea are found in invertebrates and, with respect to antitumor agents, several promising molecules have been isolated from these sea creatures, which are soft bodied and have a sedentary life style demanding chemical means of defense. This review is focused on some major classes of invertebrates’ bioactive alkaloids, useful as lead structures in the research and development of new antitumor drugs or as biological probes for physiological investigation. Their structures, origin, and significant biological activity are illustrated. Recent discoveries concerning the current state of potential and/or development of some of them as new drugs, as well as the current knowledge regarding their modes of action, are also summarized.

## 2. β-Pyridopyrrolopyrimidine Alkaloids: Variolins, a New Family of Cyclin-Dependent Kinases (CDKs) Inhibitors

**Figure 1:**
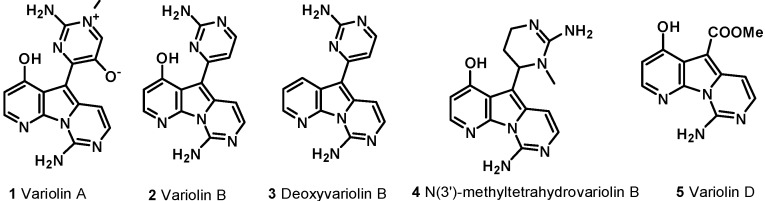
Structure of variolins.

Altered protein phosphorylation is a frequent feature in numerous human diseases; this last decade has therefore witnessed considerable efforts to identify, optimize, and evaluate pharmacologic inhibitors of protein kinases. Among the 518 human kinases, CDKs have attracted considerable interest given their involvement in many essential physiologic pathways, especially in cancer. Consequently, many pharmacologic inhibitors of CDKs have been found to display promising antitumor activities, and CDKs represent a promising anticancer drug target. Variolins constitute a new family of inhibitors of CDKs. They are marine alkaloids possessing an uncommon pyrido[3',2':4,5]pyrrolo[1,2-c]pyrimidine skeleton, which were first isolated in 1994 from *Kirkpatrickia variolosa*, a rare Antarctic sponge [1,2]. Five variolins have been isolated: variolin A (**1**), variolin B (**2**), deoxyvariolin B (**3**), N(3')-methyl tetrahydrovariolin B (**4**), and variolin D (**5**) (Figure 1). Variolin D was found to be an artifact of the extraction process (aerial oxidation of the variolins) [3]. Variolin B exhibited anticancer activity on P388 murine leukemia cells with an IC_50_ of 716 nM, whereas variolin A and N(3')-methyl tetrahydrovariolin B displayed only modest activities against this cell line, and variolin D was not active. Successively, variolin B was shown to be an efficient activator of apoptosis, showing potent cytotoxic activity against a variety of human cancer cell lines, including those overexpressing *p*-glycoprotein (*pgp*), a cell efflux pump responsible for the resistance of cancerous cells to multiple chemotherapy agents. However, further investigation of the molecular basis of variolin B activity was hampered by the limited amount of compound available from natural sources [3]. The completion of the first total synthesis of variolin B in 2001 [4] provided significant quantities of material, so that detailed studies into the mechanism of action of variolin B could begin. Cytotoxicity studies indicated that both variolin B (**2**) and its analogue deoxyvariolin B (**3**) possessed similar levels of cytotoxic activity, with both compounds exhibiting IC_50_ values of 50–100 nM against a variety of cell lines [5]. With the similar cytotoxicity of variolin B (**2**) and deoxyvariolin B (**3**) established, studies were undertaken in order to identify the molecular target of these compounds. Based on the observation that both compounds affected cell cycle progression, their ability to inhibit CDKs was investigated to understand the pivotal role of CDKs in the regulation of the cell cycle. Both variolin B and deoxyvariolin B were observed to inhibit the phosphorylation of histone H1 mediated by cyclin E-CDK2, cyclin A-CDK2, cyclin B-CDK1, cyclin H-CDK7, and cyclin D-CDK4, with IC_50_ values in the micromolar range. A preferential inhibition of CDK1 and CDK2 over CDK4 and CDK7 was observed. The inhibition of CDK1 and CDK2 is consistent with the observed cell cycle effects of the variolins. On the basis of these results, it was proposed that the primary mechanism of action of variolin B and deoxyvariolin B is to inhibit CDKs and interrupt the normal progression of the cell cycle [6]. Further understanding of the interaction between variolin B and CDKs was provided by Meijer and co-workers, who analyzed the interaction of variolin B with a variety of kinases. Inhibition of the CDKs was again observed, with the same preference of CDK1 and CDK2 inhibition over CDK4 and CDK7 inhibition. However, the inhibition of CDK9 (IC_50_ 26 nM) was determined to be even more potent than that of either CDK1 (IC_50_ 60 nM) or CDK2 (IC_50_ 80 nM). Strong inhibition by variolin B of several other kinases was observed, including casein kinase-1 (CK1), FMS-like tyrosine kinase 3, and glycogen synthase kinase-3 (GSK3) [7,8].

**Figure 2:**
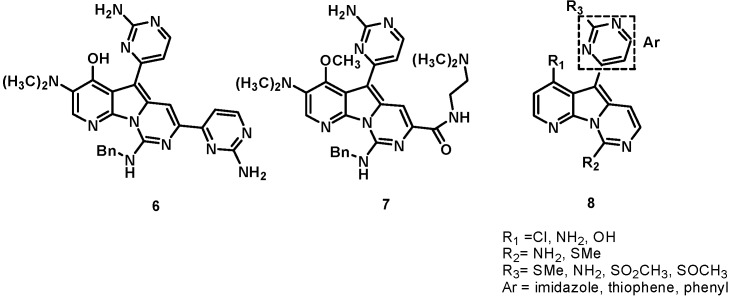
Analogs of variolins.

The unique heterocyclic framework of variolin thus emerged as a new scaffold for the design of new inhibitors of CDKs. PharmaMar performed preclinical investigations on variolins and deoxyvariolin B [9,10,11] and selected deoxyvariolin B for further development due to its favorable physicochemical properties. On the basis of the SAR results of the initial screen, a medicinal chemistry program was initiated by PharmaMar, in collaboration with Molina and Fresneda; 16 novel analogues with different substituents at positions C5 and C7 were synthesized and tested against a panel of 16 tumor cell lines [12]. From this study, two derivatives, compounds **6** and **7**, were identified that displayed activity similar to that of natural variolin B. Furthermore, PharmaMar synthesized a series of variolin analogues with general structure **8** that showed potent cytotoxicity against a panel of colon, breast, melanoma, lung, ovary, cervix, kidney, pancreas, and endothelium cell lines, with GI_50_ in the micromolar to nanomolar range [10,13]. The structures of variolin analogues are shown in Figure 2.

## 3. Indole Alkaloids: Meridianins, a New Scaffold for the Design of Novel CDK Inhibitors

**Figure 3:**
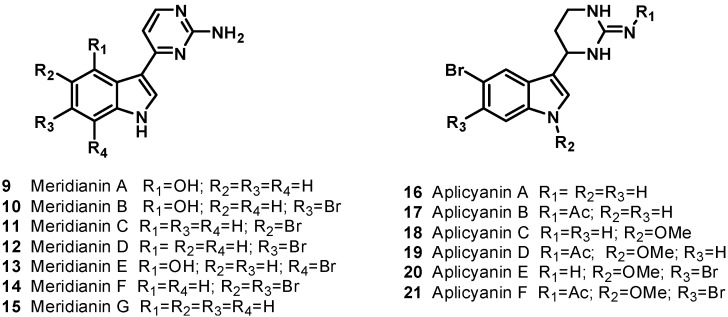
Indole alkaloids (**9**–**21**).

Meridianins are a family of 3-(2-aminopyrimidine)indoles isolated from *Aplidium meridianum*, an ascidian from the South Atlantic [14,15] that shares some structural similarity with variolins, with the meridianins possessing a pyrimidyl-substituted indole skeleton and variolin B possessing a pyrimidyl substituted pyrido[3',2': 4,5]pyrrolo[1,2-*c*]pyrimidine skeleton. Moreover, the meridianin structure was identified as a promising kinase-inhibitory scaffold [16]. The natural meridianins A–G (**9**–**15**), are brominated and/or hydroxylated 3-(2-aminopyrimidine)-indoles differing in the bromine and /or hydroxyl substitution (Figure 3). Meridianins inhibit various protein kinases, such as CDKs, glycogen synthase kinase-3, cyclic nucleotide-dependent kinases, and casein kinase 1; they also prevent proliferation and induce apoptosis probably due to their ability to enter cells and interfere with the activity of kinases important for cell division and death [14]. The unsubstituted meridianin skeleton found in meridianin G (**15**) was only weakly inhibitory, while a single bromine for hydrogen substitution at the 5- or 6- position of the indole ring (meridianin C (**11**) and D (**12**), respectively) resulted in considerable improvements in potency, with up to 1000-fold decreases in IC_50_ observed in favorable cases. Interestingly, in meridianin F (**14**), the presence of two bromine atoms, at both the 5- and 6-positions, resulted in improved potency relative to meridianin G (**15**), but decreased potency compared to either monobrominated meridianins C (**11**) or D (**12**). Substitution of a hydroxyl group for hydrogen at the indole position 4, as seen in meridianin A (**9**), resulted in smaller improvements in potency, while a bromine at position 7 and a hydroxyl group at position 4 (as seen in meridianin E (**13**)) resulted in the most potent compounds, with IC_50_ values in the low M to high nM range, depending on the target.

More recently, an additional series of compounds with a similar structure to the meridianins was discovered. The aplicyanin family was isolated from the Antarctic tunicate *Aplidium cyaneum* and consists of six variants on a core 3-(tetrahydropyrimidyl)indole structure (aplicyanins A–F, **16**–**21**, Figure 3); these alkaloids can be considered reduced forms of the relevant meridianins and, thus, their biogenetic precursors [17]. Aplicyanins B (**17**), D (**19**), and F (**21**) have been found to have significant cytotoxic activity, with IC_50_ values in the low to sub-μM range. Aplicyanins A (**16**) and C (**18**) were found to possess no cytotoxic activity at the concentrations tested, while aplicyanin E (**20**) possessed weak cytotoxic activity. These results indicate a vital role for the acetyl portion of the acetylguanidine group.

**Figure 4:**
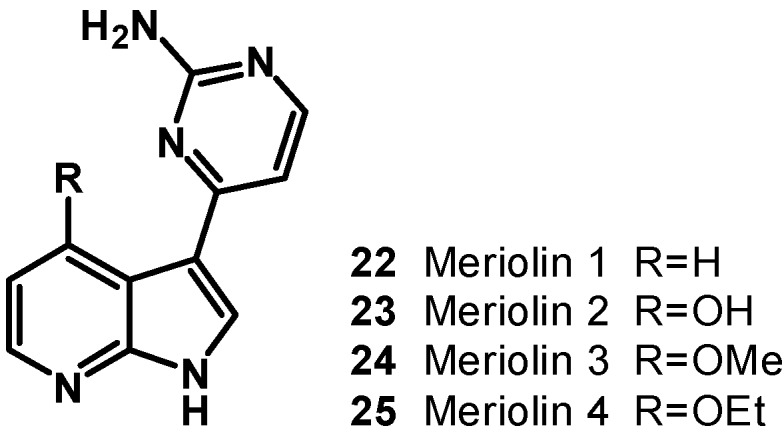
Structure of meriolins (**22**–**25**).

A new CDK inhibitory scaffold, with promising antitumor activity, has been identified by combining the common features of variolins and meridianins. A new class of 7-azaindole-containing analogues have been thus designed and the term “meriolin” has been coined to describe this hybrid structure (Figure 4) [7,8]. Meriolins (**22**–**25**) display potent inhibitory activity and relative selectivity toward CDKs and also exhibit better antiproliferative and proapoptotic properties in cell cultures than their ‘‘inspirational parent’’ molecules. Meriolins are particularly potent inhibitors of CDK2 and CDK9. The crystal structures of meriolin 3 (**24**) and variolin B in complex with CDK2/cyclin A revealed that the two molecules bind in very different orientations in the ATP-binding pocket of the kinase. Meriolins prevent phosphorylation at CDK1-, CDK4-, and CDK9-specific sites in neuroblastoma SH-SY5Y cells and induce the rapid degradation of the survival factor Mcl-1. Meriolin 3 (**24**) potently inhibits tumor growth in two mouse xenograft models, Ewing’s sarcoma and LS174T colorectal carcinoma. Meriolins thus constitute a new kinase-inhibitory scaffold with promising antitumor activity derived from molecules initially isolated from marine organisms [7].

The sponge *Topsentia genitrix*, collected from Banyuls (France) is the source of the antitumor bisindole alkaloids topsentin (**26**) and bromotopsentin (**27**), containing 2-acyl imidazole moiety inserted between two indole units with different substitution on benzene rings [18]. In 1995, Capon* et al*. reported the isolation of isobromotopsentin (**28**) from the deep water sponge *Spongosorites* sp. collected from the coast of southern Australia [19]. Topsentin (**26**) inhibited proliferation of cultured human and murine tumor cells. It exhibited* in vitro* activity against P-388 with an IC_50_ value of 3 μg/mL, human tumor cell (HCT-8, A-549, T47D) with IC_50_ value of 20 μg/mL, and* in vivo* activity against P-388 (T/C 137%, 150 mg/kg) and B16 melanoma (T/C 144%, 37.5 mg/kg) [20]. Bromotopsentin (**27**) showed antiproliferative activity against human bronchopulmonary cancer cells (NSCLC-N6) with an IC_50_ = 12 μg/mL [21]. Deoxytopsentin (**29**) was isolated from the sponge *Hexadella* sp. [22]. In 1999, bromodeoxytopsentin (**30**) and isobromodeoxytopsentin (**31**) were isolated from sponge *Spongosorites genitrix* collected from Jaeju Island, Korea [23]. Structurally, topsentin (**26**) and deoxytopsentin (**29**) are the same except for the indole ring, which is unsubstituted in the case of deoxytopsentin (**29**). Deoxytopsentin (**29**) showed the antiproliferative activity against human bronchopulmonary cancer cells (NSCLC-N6) with an IC_50_ value of 6.3 μg/mL. It also displayed moderate activity against breast cancer and hepatoma (HepG2) with an IC_50_ of 10.7 and 3.3 μg/mL, respectively.

**Figure 5:**
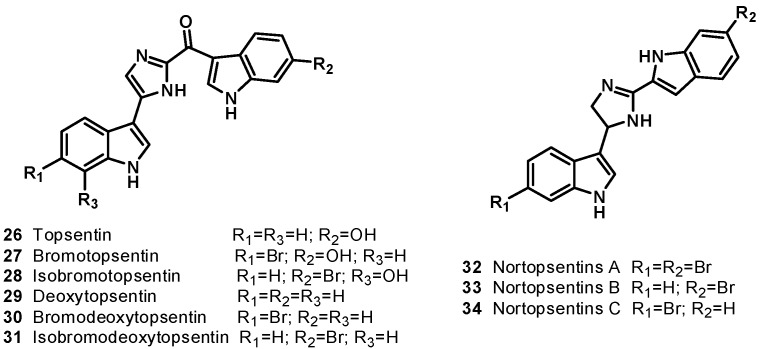
Bis indole alkaloids **26**–**34**.

**Scheme 1:**
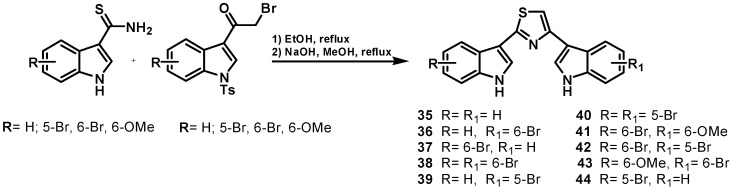
Synthesis of indolylthiazole compounds **35**–**44**.

Nortopsentins A (**32)**, B (**33)**, and C (**34)** (Figure 5), having a characteristic 2,4-bis(3-indolyl)imidazole skeleton, were isolated from the deep water marine sponge *Spongosorites ruetzleri* [24]. Nortopsentins A–C exhibited* in vitro* cytotoxicity against P388 cells: IC_50_ (μg/mL), 7.6, 7.8 and 1.7, respectively [25,26]. Indolylthiazole compound **35**–**44** analogs (Scheme 1) of nortopsentins were synthesized and evaluated for cytotoxicity in the NCI’s* in vitro* disease-oriented antitumor screen against a panel of approximately 60 human tumor cell lines derived from leukemia, non-small-cell lung cancer, colon cancer, CNS cancer, melanoma, ovarian cancer, renal cancer, prostate cancer, and breast cancer [27]. Compounds **35**–**44** all exhibited cytotoxic activity against a variety of human cancer cell lines. Compound **35** selectively exhibited* in vitro* cytotoxicity against leukemia and ovarian cancer cell lines, affording a GI_50_ of 3.27 μM in K562, 5.31 mM in Molt-4, and 8.14 μM in an IGROV1 assay. In the other human tumor cell line assay, the GI_50_ of compound **35** exceeded 100 μM. To test the possibility that substitutions in the indole ring might result in a potency increase, most of the 2,4-bis(3-substituted-indolyl)thiazoles showed broad effects on tumor cell lines from leukemia, colon cancer, CNS cancer, and breast cancer panels, while unsubstituted counterpart **35** brought out highly selective activity against leukemia cell lines and IGROV1 ovarian cancer cell lines. The position of bromine in the indole ring plays an important role in cytotoxicity. Dibrominated compound **44** effectively exhibited MCF 7 in breast cancer, affording a GI_50_ of 0.888 μM [28].

## 4. Bis-Steroidal Pyrazines: Ritterazines and Cephalostatins, Potent Inducers of Apoptosis Acting via a Novel Antimitotic Mechanism

Ritterazines are highly oxygenated dimeric steroidal alkaloids isolated by Fusetani and co-workers from 1994 to 1997 from the colonial tunicate *Ritterella tokioka* (family Policlinidae). Together with cephalostatins, isolated from the marine worm *Cephalodiscus gilchristi*, they make up a unique family of 45 trisdecacyclic bis-steroidal pyrazines that display extremely potent cytotoxicity against human tumors with unique cell selectivity and apoptotic response [29,30,31]. The outstanding antineoplastic potency, together with the new and challenging molecular architecture, made these marine invertebrate alkaloids promising anticancer leads.

**Figure 6:**
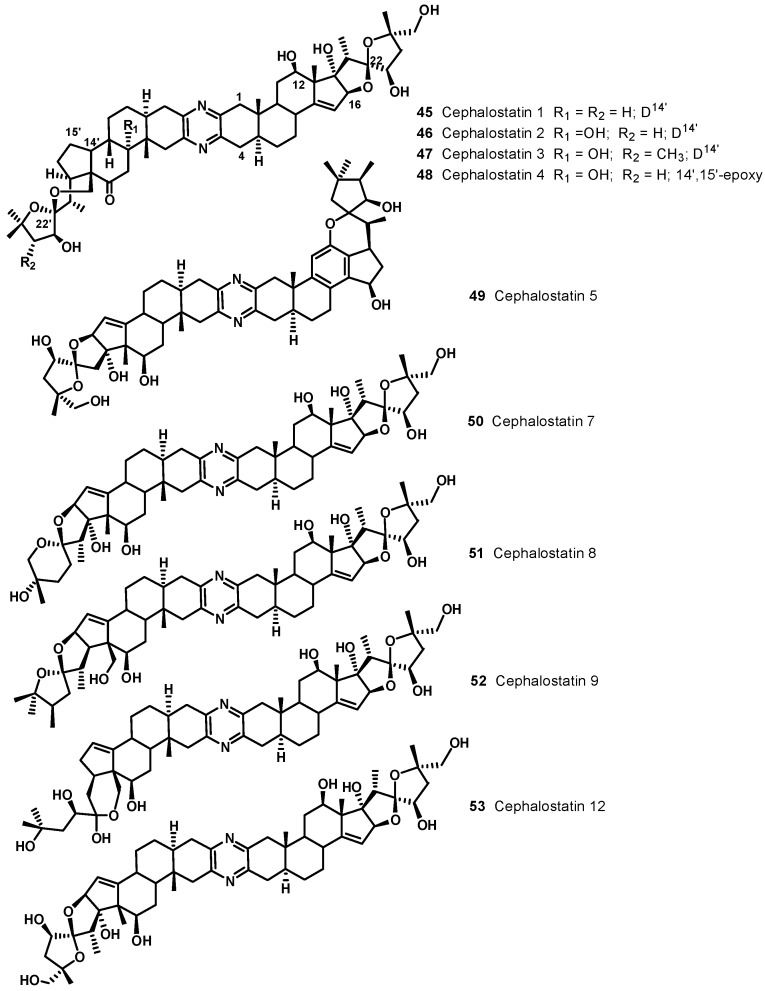
Selected cephalostatin structures.

**Figure 7:**
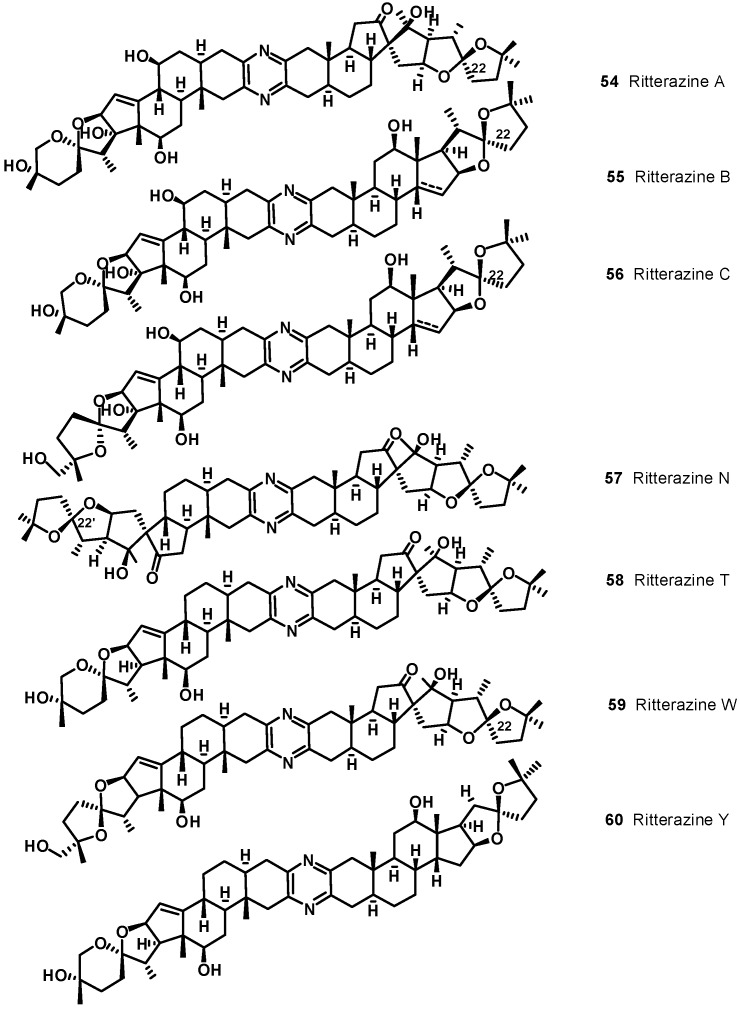
Selected ritterazine structures.

Illustrative examples of cephalostatin and ritterazine structures are reported in Figure 6 and Figure 7, respectively. The ritterazines and cephalostatins share many common structural features. Both consist of two highly oxygenated C27 steroid units fused via a pyrazine ring at C-2 and C-3; both chains of the steroid units usually form either 5/5 or 5/6 spiroketals. While cephalostatins in general are more oxygenated on the right side, the ritterazines have the more oxygenated left side. Hydroxyl groups are present at C-12, C-17, C-23, C-26, C-12′, and C-23′ in the cephalostatins, whereas C-12, C-7', C-12', C-17', and C-25' are hydroxylated in the ritterazines. Cephalostatins 5 (**49**) and 6 are unusual in that they both contain an aromatic C ring. While naturally occurring and synthetic steroids with aromatic A rings are well-known, steroids bearing an aromatic C ring are quite rare. Cephalostatin 12 (**53**) is the only symmetric constituent (right unit = left unit). Ritterazines N (**57**)-S, having two non-polar steroidal units, were much less active than ritterazine B. Ritterazines T (**58**)-Y are related to ritterazines A (**54**) and B (**55**), composed of polar and non-polar steroidal units, lacking the C7' and C17' hydroxyl groups. Ritterazine Y (**60**) differs from ritterazine B in the absence of the two hydroxyl groups, whereas ritterazines W and X have the 5/5 spiroketal terminus in the polar steroidal unit instead of the 5/6 spiroketal terminus. In ritterazines T and V, both steroidal units are rearranged.

Cephalostatin 1 (**45**) is among the most powerful anticancer agents ever tested, displaying subnanomolar-to-picomolar cytotoxicity against much of the National Cancer Institute’s (NCI) 60-cell line panel, with femtomolar activity against the P388 cell line and in the Purdue Cell Culture Laboratory (PCCL) human tumor panel [29,30]. Most of the remaining cephalostatins were similarly potent. A dramatic reduction in PS cell growth inhibition was displayed by cephalostatins 5 and 6, suggesting that preservation of structural integrity in the right-hand side unit, including C/D ring stereochemistry, is very important to realizing powerful cytotoxicity. Cephalostatin 12 also displayed significantly reduced inhibitory activity in comparison to other cephalostatins, suggesting that asymmetry is necessary for optimum cytotoxicity [29]. Ritterazines are less potent than cephalostatins, with P388 inhibitory activities ranging from 3.6 μM for ritterazine W (**59**) to 0.17 nM for ritterazine B (**55**) (see Table 1), the most active constituent of *R. tokioka*, which, not surprisingly, contains nearly the same right-hand side steroid unit (**70**: no 17*R*-hydroxy moiety) as the most active cephalostatins (**45**–**48**, **50**–**52** see Table 1).

The 45 members of the cephalostatin/ritterazine family isolated to date, together with a growing number of analogues and related monosteroidal antineoplastics provide the basis for elucidating some structure–activity relationships (SAR) of these potent cytotoxins and for discovering the minimum pharmacophore required to maintain potent cancer cell growth inhibitory behavior. These studies suggest that four features conspire to provide active* in vitro* materials: (i) a molecular dipole consisting of covalently linked lipophilic “nonpolar” and hydroxylated “polar” domains, with a molecular length of ~30 Å; (ii) a spiroketal or other latent precursor of an oxocarbenium ion; (iii) one or more homoallylic oxygen arrays; and (iv) a 17-OH function. The pyrazine ring, though present in most examples, is absent in several subnanomolar active monosteroids [30].

**Table 1:** Murine P388 lymphocytic leukemia inhibitory activity of ritterazines and cephalostatins.

Ritterazine	(ED_50_ nM)	Cephalostatin	(ED_50_ nM)	Ritterazine	(ED_50_ nM)	Cephalostatin	(ED_50_ nM)
**A**	l4.2	**1**	10^−4^–10^−6^	**N**	522	**14**	4.4
**B**	0.17	**2**	10^−4^–10^−6^	**O**	2383	**15**	26.2
**C**	102.3	**3**	10^−4^–10^−6^	**P**	819	**16**	<1.1
**D**	17.5	**4**	10^−4^–10^−6^	**Q**	657	**17**	4.4
**E**	3.8	**5**	42.5	**R**	2461	**18**	4.6
**F**	0.81	**6**	2.3	**S**	539	**19**	7.9
**G**	0.81	**7**	1–<0.1	**T**	522		
**H**	17.8	**8**	1–<0.1	**U**	2341		
**I**	15.3	**9**	1–<0.1	**V**	2341		
**J**	14.0	**10**	3.2	**W**	3631		
**K**	10.4	**11**	2.7	**X**	3404		
**L**	11.1	**12**	76.2	**Y**	4.0		
**M**	16.7	**13**	47.9	**Z**	2200		

A COMPARE pattern-recognition analysis gave correlation coefficients of ~0.9 between cephalostatin 1 and ritterazine B in NCI-10 cell lines, suggesting that ritterazines act by the same mechanism as do cephalostatins; this mechanism, however, is presently largely unknown [32]. The fingerprint of ritterazines’ activity in the NCI 60-tumor panel is quite different from known anticancer agents, likely indicating a new mechanism of action. Bis-Steroidal pyrazine alkaloids do not contain functional groups commonly associated with cytotoxicity such as alkylation sites, Michael acceptors, intercalators, or redox-active quinones. Early speculation on the mode of action of these compounds centered around the likelihood of cell membrane penetration due to their steroidal nature and dimensions or, alternatively, the possibility that the compounds serve as a spatially defined set of hydrogen-bond donors/acceptors for enzyme binding [33]. Proapoptotic properties have been demonstrated for ritterazine B (55), although apoptosis induced by this compound appeared to be independent of the caspase pathway [32]. A recent biological study clearly demonstrated that cephalostatin 1 (45) evokes a new cytochrome c-independent apoptosis signaling pathway [34]. This is in contrast to most of the well-known anticancer drugs, which act in a cytochrome c-dependent route, showing that ritterazine/cephalostatin alkaloids family might be potent inducers of apoptosis, acting via a novel antimitotic mechanism, implying that they could be used to treat drug-resistant cancers [32,34,35,36].

Despite the number and value of synthetic contributions to the cephalostatins and ritterazines [29,30], owing to the complexity of the targets, only very small amounts of these substances were produced and in very low overall yields, not suitable to supply sufficient samples at reasonable cost for extended biological evaluation. The availability of these compounds from their only known natural sources, the marine worm *C. gilchristi* and the marine tunicate* Ritterella tokioka*, is still extremely limited; while the isolation yields of the ritterazines are slightly better than the cephalostatins, they also are too low to supply clinical trials. As a result,* in vivo* anticancer evaluation of these very promising natural products and subsequent preclinical development have been greatly restricted. Nevertheless, the occurrence in different phyla of cephalostatins and ritterazines, closely related in structure metabolites, raises questions as to their true production and may indicate a microbial origin for this family of compounds [29,30]. The current good possibility that cephalostatin/ritterazine alkaloids inhibit cancer cell growth by affecting a novel molecular target(s), the ongoing total synthetic and SAR challenges, the possibility of locating a marine microorganism source actually responsible for their biosynthesis, and clinical development prospects all suggest that the bis steroidal alkaloids field will become increasingly productive and useful.

## 5. Dopa [2-Amino,3-(3',4'-dihydroxyphenyl) Propionic Acid] Derived Pyrrole Alkaloids: Lamellarins, Potent Inhibitors of Topoisomerase (TOPO) I with Multi-Drug Resistance (MDR) Modulating Activity

Among the most promising anti-cancer drug candidates derived from marine invertebrates is the family of lamellarins [37,38,39,40,41]. The lamellarins form a group of more than 50 highly condensed DOPA- derived pyrrole alkaloids that have attracted researchers’ interest due to both their structural originality and significant biological properties. Lamellarins were originally isolated from the prosobranch mollusk *Lamellaria* sp. in 1985 [42]. They were later extracted from various ascidians belonging to the *Didemnum* genus [43,44,45,46,47,48] and in some sponges [49,50] collected from widely varying locations, which suggests a potential microbial link to their biosynthesis.

**Figure 8:**
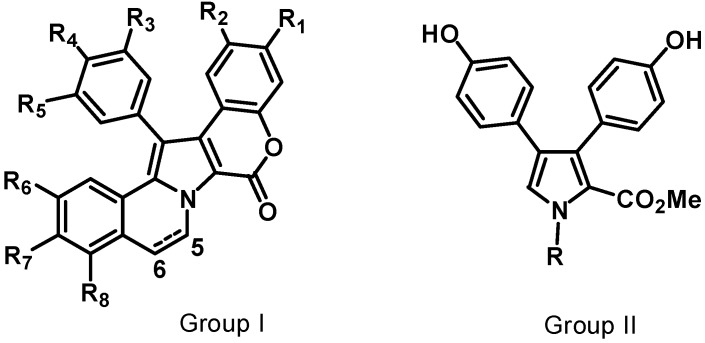
Core structures of lamellarins.

**Figure 9:**
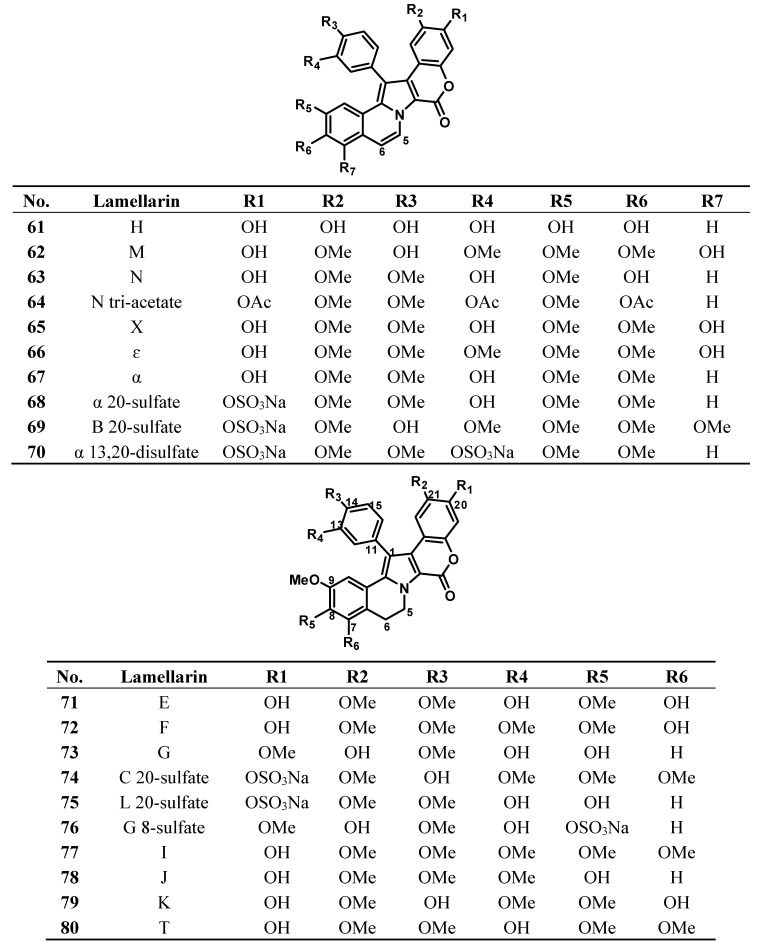

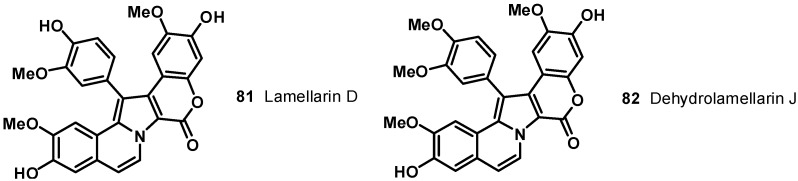
Selected structures of lamellarins isolated from ascidians (**61**–**82**).

The lamellarins have a pyrrole ring as a core component of their skeleton. They fall into two structural groups, depending on whether the central pyrrole ring is fused (Group I) or unfused (Group II) to adjacent aromatic rings. Group I could be further divided into two subgroups, Ia, including compounds possessing an olefin at C5/C6, and Ib, with compounds in which this olefin is saturated (Figure 8). Each group includes derivatives in which phenolic hydroxyl groups are substituted by methoxy, sulfate, or acetate functions. The lamellarins isolated from ascidians possess the hexacyclic skeleton of Group I; selected structures of ascidians’ lamellarins (compounds **61**–**80**) are reported in Figure 9.

The most common and remarkable property of the lamellarins is their capacity to inhibit the proliferation of cancer cells [51,52,53]. The majority of lamellarins are considerably cytotoxic, with IC_50_ (or LD_50_) values in the nanomolar to micromolar range, depending on the experimental conditions and the nature of the compounds. A noticeable exception is the sulphated lamellarins, which are not cytotoxic presumably due to reduced cell uptake. Lamellarins D (**81**), K (**79**), and M (**62**), are the most potent compounds in the series [38], but lamellarin N (**63**) and dehydrolamellarin J (**82**) are also promising candidates.

Following the discovery of the potent anti-proliferative and proapoptotic activities of lamellarins, their biological activities have been extensively studied, in particular their capacities to interfere with topoisomerase (TOPO) I and mitochondria, both contributing to their potent cytotoxicity. Although all the aspects of the lamellarins’ mechanism of action are not completely known, it has been demonstrated that they are potent inhibitors of topoisomerase (TOPO) I, they interact with DNA, they target mitochondria directly, and they induce the release of cytochrome C and apoptosis-inducing factor (AIF) [51,53,54,55,56,57,58]. Lamellarin D (**81**) was identified as a potent TOPO I poison in 2003 [53]; it binds relatively weakly to DNA, presumably via the insertion of its planar pentacyclic chromophore between DNA base pairs. Intercalation of the flat chromophore between two adjacent base pairs exposes the perpendicular methoxyphenol moiety toward the major groove of DNA where the enzyme can be trapped [54,55]. This DNA interaction, although relatively weak, provides the necessary anchorage for stabilization of the enzyme-DNA complex. Inhibition of topoisomerase I by lamellarin D has been demonstrated by a variety of approaches, both* in vitro* (using recombinant enzymes and model DNA substrates) and in a cellular context, using immunoblot assays to detect the drug-stabilized topoisomerase I–DNA complexes in cells [40,41].

Cytotoxicity of lamellarin-D cannot be explained uniquely on the basis of TOPO I targeting. Early studies on the mechanism of action of lamellarin D already suggested the existence of alternative target(s) for this molecule in cancer cells [53]. This hypothesis was rapidly validated with the discovery of a nuclear TOPO I-independent, direct effect on mitochondria. Studies on the apoptotic pathway induced by lamellarin D revealed a very early mitochondrial dysfunction, including a reduction of mitochondrial membrane potential and the release of cytochrome c and AIF from the mitochondria to the cytosol [57,58]. It was then observed that lamellarin D could induce these alterations directly on isolated mitochondria. Both functional assays and direct observations by electron microscopy indicated that mitochondria represented targets for lamellarin D, and similarly for lamellarin M (**62**) [40].

Some lamellarins also function as multi-drug resistance (MDR) reversal drugs [51,56]. MDR is a term used to characterize the ability of tumors to exhibit simultaneous resistance to various chemotherapeutic agents through diverse mechanisms but, more frequently, through modification of drug efflux implicating ABC transporters, such as the multidrug resistance-associated protein (MRP) or P-glycoprotein (Pgp). In tumor cells expressing Pgp, the efflux of a given anticancer drug increases across the plasma membrane, thereby reducing the intracellular drug concentration and hence its cytotoxicity; Pgp activity can be reversed by a few specific molecules, referred to as MDR modulators. Lamellarins I (**77**), K (**79**), and T (**80**) have been reported to act as MDR modulators; these compounds exhibited equally effective cytotoxic activity against MDR cell lines and demonstrated that even at non-cytotoxic concentration they reverse MDR by inhibiting P-glycoprotein-mediated drug efflux [56]. It has been demonstrated that lamellarin I (**77**) reversed MDR by directly inhibiting the P-glycoprotein-mediated drug efflux at nontoxic doses and that the potency of lamellarin I as an MDR modulator was 9–16-fold higher than that of approved anticancer agent verapamil, resensitizing the resistant malignant cells to frontline therapeutics [51].

Elegant synthetic routes to lamellarins or their key intermediates have been developed, which afforded sufficient quantities of structurally simplified lamellarin analogs for SAR and other biological studies [39,41]. In an early attempt to correlate the structures of lamellarins with their cytotoxic activities, Quesada* et al*., observed that an increase in the number of methylations and/or methoxylations appears to cause a decrease in the antitumor activities of the 13 lamellarins tested in their studies [51]. Most subsequent SAR studies have been directed at derivatives of lamellarin D, also by using their mechanism-based (*i.e*., TOPO I inhibition) activities in relation to their cytotoxicities and precise structure-activity relationships have been delineated. It has been demonstrated that the full pentacyclic structure of lamellarins is important for their biological activity. Simplification of the lamellarin D structure by opening the lactone ring results in a significant decrease in cytotoxicity toward certain human tumor cell lines. Most of the lamellarin D derivatives with an open lactone ring were found to be considerably less cytotoxic than lamellarin D, except when a lactonization potential is preserved. In this case, a marked toxicity toward A-549 lung carcinoma, HT-29 colon carcinoma, and MDA-MD-231 breast adenocarcinoma cells was maintained [59]. Another general observation is that the planarity of the pharmacophore conferred by the double bond between carbons 5 and 6 in the quinoline B-ring is a crucial element for cytotoxicity and TOPO I inhibition; when this double bond is lacking, as in lamellarin K (**79**), for example, the planar conformation no longer exists and the drug loses its capacity to interfere with TOPO I. This was clearly demonstrated using pairs of lamellarins, each of which contains exactly the same substituents and differs only in the nature of the C5/C6 bond, where the presence of the C5/C6 double bond significantly decreases the IC_50_ values [40,53,54,60].

The hydroxyl groups at the C8 and C20 positions of lamellarin D are important structural requirements for cytotoxic activity, whereas neither the hydroxyl group at C14 nor the two methoxy groups at C13 and C21 are necessary. However, it has been subsequently reported that all of the phenolic hydroxyl groups at each of the C8, C14, and C20 positions of lamellarin D are important for maintaining activity against TOPO I and potent cytotoxic action. It has been also found that these groups could be substituted with positively charged amino acid derivatives without loss of activity [54]. Furthermore, it has pointed out that almost any modifications of the substitution pattern on lamellarin D decrease the cytotoxicity of the molecule [38,60]. Substitution of the hydrogen atom at C7 with a hydroxyl group significantly increases the cytotoxicity of lamellarins with a C5/C6 double bond (compare lamellarin α (**67**) and lamellarin X (**65**)). On the other hand, methoxylation at this position may only slightly affect the cytotoxic activities of these compounds. Interestingly, the effect is significantly more pronounced if the C7 hydroxyl group is replaced by a methoxyl group. This clearly decreases the cytotoxic activities of the lamellarins, especially those containing a C5/C6 double bond. These results indicate that the hydroxyl group at this position is an important structural element that may also feature in an interaction with the putative biological target(s) [60]. Recently Khiati* et al*., investigated the effects of lamellarin D on mitochondrial topoisomerase I (Top1mt) using* in vitro* and* in vivo* DNA cleavage assays in addition to single-molecule supercoil relaxation measurements. They found that Lam-D inhibits Top1mt by trapping the cleavage complex efficiently, in striking contrast to the inefficacy of camptothecin. This study provides evidence that Top1mt is a direct mitochondrial target of Lam-D and suggests that developing Top1mt inhibitors represents a novel strategy for targeting mitochondrial DNA. Furthermore, this study demonstrates that the marine alkaloid lamellarin D is the first drug to target mitochondrial DNA by trapping Top1mt cleavage intermediates (Top1mt cleavage complexes) and suggests that developing Top1mt inhibitors could be an alternative strategy for targeting mitochondrial DNA [61].

Recognition of the biological potential of this large class of alkaloids can be found in recent reports of lamellarins entering into preclinical development for the treatment of multidrug-resistant tumors. More than 300 derivatives have been synthesized by chemists at PharmaMar (Madrid, Spain) and drug candidates have been selected for the extended preclinical and toxicological studies required prior to human clinical trials [62].

## 6. Tetrahydroisoquinoline Alkaloids: Ecteinascidins, a New Class of DNA Binding Agents

The tetrahydroisoquinoline alkaloids ecteinascidins, isolated mainly from the Caribbean ascidian *Ecteinascidia turbinata*, are probably the most useful anticancer agents found to date in a marine source [63,64]. The lead compound, trabectedin (ET-743, **84**), is regarded as a successful story of modern marine drug research: it is indeed the first representative of a marine natural product to receive marketing authorization for the treatment of patients with advanced or metastatic soft tissue sarcomas (STS) and relapsed platinum-sensitive ovarian cancer under the brand name Yondelis^®^ [65,66].

**Figure 10:**
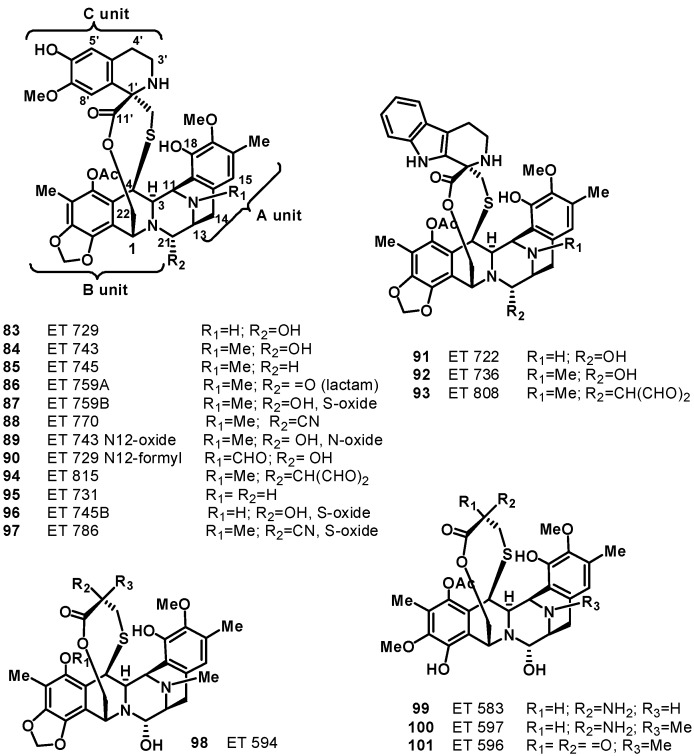
Structures of ecteinascidins (**83**–**101**).

The first description and structural characterization of six new chemical entities called ecteinascidins, ET 729 (**83**), ET 743 (**84**), ET 745 (**85**), ET 759A (**86**), ET 759B (**87**), and ET 770 (**88**), was reported by the Rinehart group in 1990, of which ET-743 was the most abundant representative [67]. Simultaneously, Wright and co-workers described compounds **83** and **84** [68], but the unequivocal assignment of the absolute stereochemistry was achieved only when the X-ray crystal structures of the natural *N^12^*-oxide of ET-743 (**89**) and a synthetic O-methyl analogue of *N^12^*-formyl ET-729 (**90**) were solved [69]. Successively, a number of additional new members of this class of molecules have been isolated, such as compounds **91** and **92** [70], **93**–**96** [71], **97** [72], and **98**–**101** [73] (Figure 10).

Apart from the biological activity, ecteinascidins exhibit an extraordinary three-dimensional molecular architecture; their unique structure consists of a monobridged pentacyclic skeleton composed of two fused tetrahydroisoquinoline rings (subunits A and B) linked to a 10-membered lactone bridge through a benzylic sulfide linkage. Most ecteinascidins have an additional tetrahydroisoquinoline or tetrahydro-β-carboline ring (subunit C) attached to the rest of the structure through a spiro-ring (Figure 10). The carbon and nitrogen framework of units A–B of the ecteinascidins is the same as that of the saframycin, safracin, and renieramicin families of antitumor agents isolated from bacteria and sponges [74]. It has been proposed that A–B units could be formed by condensation of two DOPA-derived building blocks, and the tetrahydroisoquinoline ring in unit B is closed by condensation with a serine- (or glycine-) derived aldehyde as in the case of the related saframycins. S-Adenosylmethionine is the likely source of methyl groups at C-6, O-7, C-16, O-17, and N-12 [74].

Initial structure-activity relationships of natural members of ecteinascidins were established based on results from an* in vitro* cytotoxic screening assay against murine leukemia cells L1210. These data revealed that the carbinolamine of C-21 is crucial for optimal bioactivity and the presence of the aromatic C subunit was found to be important for potency. Later, these compounds were shown to possess strong* in vivo* antitumor effects in various mice models bearing P388 lymphoma, B16 melanoma, M5076 ovarian sarcoma, Lewis and lX-1 human lung carcinoma, and MX-1 human mammary carcinoma xenografts [70]. ET-743 (trabectedin, **84**) was selected for further development; the classical NCI60 human tumor cell lines anticancer drug screen revealed that the drug was several orders of magnitude more potent than other anticancer agents with prominent growth inhibition in the pM to low nM dose range, whereas no correlation with any other known drugs was detected by COMPARE analysis, indicating that trabectedin has a unique mode of action. Subsequent studies revealed it as the first of a new class of DNA-binding agents with a complex, transcription-targeted mechanism of action. In has been demonstrated that units A and B bind covalently and reversibly to the DNA minor groove, with preference for GC-rich triplets; subsequently, trabectedin forms covalent adducts with the N2-position through its carbinolamine moiety. This induces DNA bending towards the major groove. The third unit, C, being unbound, could interact with nuclear proteins. The transcriptional activation of inducible genes is inhibited depending on the presence of the DNA repair systems, particularly the transcription-coupled nucleotide excision repair (TC-NER) system. In the absence of trabectedin, TC-NER recognizes and removes DNA lesions from transcribed strands of expressed genes. This is followed by removal of the DNA segment containing the lesion and gap polymerization using the intact strand as a template. In the presence of trabectedin, the TC-NER machinery is recruited into the DNA adduct region in an attempt to correct the DNA lesion but is blocked, creating strong cytotoxic complexes that will induce double strand DNA breaks and cell death. Thus, while all other known DNA-interacting agents require a deficient NER mechanism to exert their activity, trabectedin needs a proficient NER system to exert its cytotoxic activity [75,76,77,78,79].

Trabectedin was tested in a great variety and number of models against tumors of murine origin and human sensitive and resistant xenografts and showed a broad spectrum of antineoplastic activity. The observation that STS appeared more sensitive to the drug than other solid tumors came from Phase I clinical trials and the clinical benefit of trabectedin in STS patients was subsequently confirmed in Phase II clinical trials. Trabectedin, developed by the Spanish pharmaceutical company PharmaMar under the trade name Yondelis^®^, was approved for the treatment of refractory soft-tissue sarcomas by the European Commission in July 2007; the currently ongoing Phase III trials along with the already existing clinical evidence may provide enough data for the Food and Drug Administration to issue an approval in the US [80,81,82]. In November 2009 Yondelis^®^ received its second marketing authorization from the European Commission for the treatment of relapsed platinum-sensitive ovarian cancer in combination with DOXIL^®^/Caelyx^®^. Phase II trials with Yondelis^®^ are also being carried out for breast cancer and for pediatric tumors. Another Phase III trial is ongoing for soft tissue sarcoma in first line of treatment [83].

**Figure 11:**
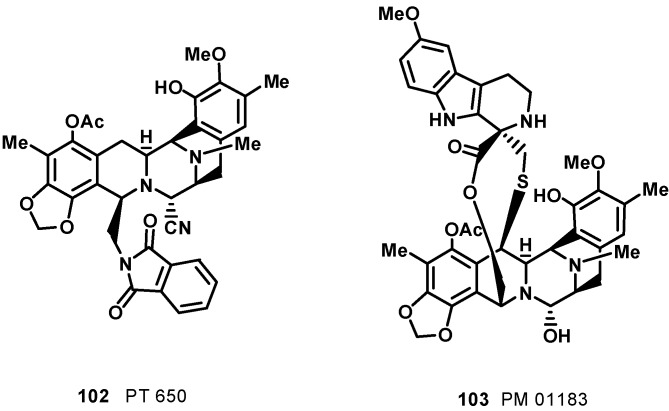
Structures of ET 743 synthetic analogs phtalascidin (PT 650, 102) and lurbinectedin (PM 01183, 103).

As is usual in marine natural products development, obtaining sufficient amounts of ET743 has been a significant challenge due to its restricted natural availability (1 g from 1 ton of tunicate). Methods of producing ecteinascidins by in-sea culture of *E. turbinata* have been evolved [84], and much synthetic effort has been directed towards the synthesis of ecteinascidins. The first total synthesis of ET-743 was published by the Corey group in 1996 [85]. The synthesis was inspired, at least in part, by the proposed biosynthesis of the natural product, which is believed to involve the dimerization of two tyrosine residues to form the pentacyclic core of the molecule, and was based on biomimetic disconnections leading to four ‘‘amino acid’’ subunits. The synthesis uses such reactions as the Mannich reaction, the Pictet–Spengler reaction, the Curtius rearrangement, and chiral rhodium-based diphosphine-catalyzed enantioselective hydrogenation. A separate synthetic process also involved the Ugi reaction to assist in the formation of the pentacyclic core. This reaction was unprecedented for using such a one-pot multi-component reaction in the synthesis of such a complex molecule. Further synthetic approaches have been reported by a number of research groups [86,87,88,89,90,91]; notable examples are the total synthesis accomplished by Fukuyama* et al.*, [87] and Zhu *et al.*, [88]. However, both aquaculture of the tunicate and total synthesis cannot provide economical access to the drug. Currently, ET-743 for clinical application is produced by a semi-synthetic process in 17 chemical steps developed by PharmaMar, starting from cyanosafracin B, an antibiotic produced by the fermentation of *Pseudomonas fluorescens* [92]. The semisynthetic approach provides access also to other natural members of the ecteinascidin family, such as ET-729 (**83**), ET-745 (**85**), ET-759B (**87**), ET-736 (**92**), and ET-594 (**98**) [93]. The desirability of finding simpler and more stable structural relatives of ET 743 has led researchers to design, synthesize, and evaluate new members of the class; among them, phtalascidin (PT 650, **102**, Figure 11) displayed an antitumor profile and activity comparable to ET 743 and was more readily synthesized and more stable than ET 743 [94,95,96]. PM01183 (lurbinectedin, **103**, Figure 11) is another very promising synthetic analog of ET 743; it is structurally similar to ET 743 except for the C subunit, where the tetrahydroisoquinoline present in ET 743 is replaced by a tetrahydro β-carboline in PM01183 [97]. This structural variation is accompanied by important modification of pharmacokinetic and pharmacodynamic properties in cancer patients [75,98]. Like ET743, PM01183 covalently binds to the minor groove of the DNA-forming DNA adducts, which give rise to double strand breaks and perturbations of the cell cycle inducing cell death. In preclinical studies, the compound displays a potent cytotoxic activity against tumor cell lines of different origin. It is currently in Phase II clinical trials for relapsed ovarian, lung, breast, and pancreatic cancer. PM01183 is also undergoing Phase I development in combination with other chemotherapies and in hematological tumors. On August 20, 2012, PharmaMar received FDA orphan drug designation (ODD) for PM01183 for the treatment of ovarian cancer [98,99].

## 7. Pyridoacridine Alkaloids: DNA Intercalating Agents with Reactive Oxygen Species (ROS) Producing and TOPO-Inhibiting Activities

**Figure 12:**
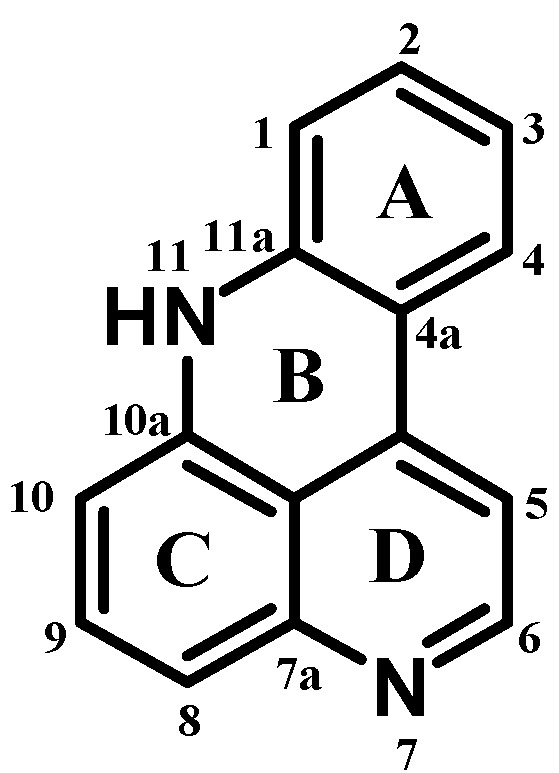
General structure of pyridoacridines.

Pyridoacridines are a class of strictly marine-derived alkaloids that constitute one of the largest chemical families of marine alkaloids, which share an 11*H*-pyrido[4,3,2-*mn*]acridine skeleton (Figure 12) [100].

Pyridoacridine alkaloids display diverse biological activities including cytotoxicity, production of reactive oxygen species (ROS), and topoisomerase inhibition. These activities are often dependent on slight modifications to the pyridoacridine skeleton. The first member of this family was calliactine, which was isolated from the marine anemone *Calliactis effoeta* in 1940 [101]. Over 40 years later, the second member, amphimedine, was found from the marine sponge *Amphimedon* sp. [102]. Since then, more than 80 pyridoacridines have been found in many marine organisms such as sponges, tunicates, anemones, and mollusks [100,103,104]. Most pyridoacridines have been reported to have significant cytotoxicity due to a highly planar electron-deficient aromatic ring system that can intercalate DNA, resulting in the inhibition of cell growth [105]. Besides possessing a generally nonselective mechanism of cytotoxicity, pyridoacridines also have certain specific biological properties in living systems, including antibacterial, antifungal, antiviral, and antiparasitic activities [106,107,108,109,110]. These compounds show anticancer effect: they inhibit TOPO II and aspartate semialdehyde dehydrogenase, produce reactive oxygen species, cause the release of calcium from the sarcoplasmic reticulum, induce neuronal differentiation, and bind nucleotide receptors [105,111]. Structurally, pyridoacridines are highly-colored marine natural products with a polycyclic planar heteroaromatic 11*H*-pyrido[4,3,2,*mn*]acridine system [103]. The structure of pyridoacridines presents a large structural variety; a very interesting pyridoacridine family tree has been created to analyze the biodiversity in this rich family of marine compounds [112].

During the last few years, numerous additional compounds of this family were isolated; most of them are polycyclic with different substituents such as shermilamine, kuanoniamine, neoamphimedine, arnoamines, and styelsamines. It has been observed that almost all the pyridoacridines show promising cytotoxicity against different type of tumors. Therefore, interest grew in modifying the pyridoacridine moiety for developing a new generation of therapeutic agents. Research indicates that DNA binding, the inhibition of DNA metabolizing enzymes, and the production of ROS may all play a role in the cytotoxicity of the pyridoacridines [105].

Pyridoacridines vary structurally by attachment of different side chains or fusion of different rings to ring C of the basic structure (Figure 12) and less often to the acridine nitrogen. Halogen substitution in pyridoacridines is quite rare; even if it is present, then it is always bromine at C2 in ring A. Oxidation states of the rings are variable and in some cases ring D is partially saturated. Additional rings are often attached to ring C [113].

**Figure 13:**
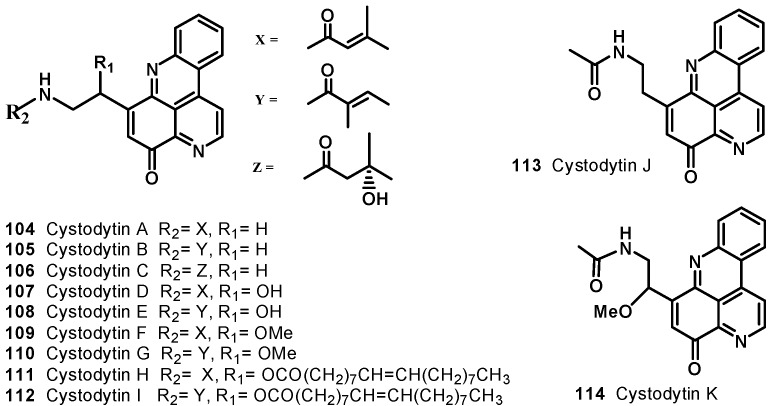
Structures of cystodytins A–K (**104**–**114**).

Nine cytotoxic tetracyclic alkaloids, cystodytins A–I (**104**–**112**) have been identified from yellow tunicate *Cystodytes dellechiajei* (Figure 13) [114,115]. Compounds **104**, **105**, and **106** showed potent cytotoxicity against L-1210 with IC_50_ values of 0.22, 0.22, and 0.24 μg/mL, respectively. Cystodytins D-I (**107**–**112**) were also found to be cytotoxic against murine lymphoma L-1210 cells, with IC_50_ values of 1.1 (**107** and **108**), 0.068 (**109** and **110**), and 0.080 (**111** and **112**) μg/mL, and values of 1.4 (**107** and **108**), 0.078 (**109** and **110**), and 0.092 (**111** and **112**) μg/mL against human epidermoid carcinoma KB cells* in vitro*.

Cystodytin J (**113**), isolated from ascidian *Cystodytes* sp. [116], showed cytotoxic activity against HCT and xrs-6 with IC_50_ values of 1.6 and 135.6 μM, respectively. It also inhibited the topoisomerase (TOPO) II-mediated decatenation with IC_50_ value of 8.4 *μ*M. Appleton* et al*., reported isolation of cystodytin K (**114**), a 12-methoxy derivative of cystodytin J (**113**) (Figure 13), from the extract of an ascidian *Lissoclinum notti* collected near Leigh Harbour, Northland, New Zealand [117]. Cystodytin K (**114**) exhibited cytotoxic activity against a P-388 murine leukemia cell line with an IC_50_ value of 1.3 μM.

**Figure 14:**
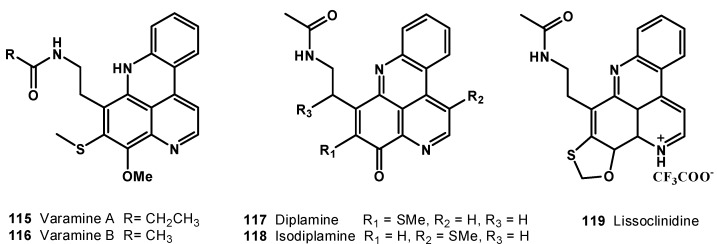
Structures of pyridoacridine alkaloids (**115**–**119**).

Two bright crimson pigments, varamine A (**115**) and B (**116**) (Figure 14), were isolated from the Fijian ascidian *Lissoclinum vareau* [118]. Varamine A (**115**) and B (**116**) exhibited cytotoxicity towards L-1210 murine leukemia cells with IC_50_ values of 0.03 and 0.05 μg/mL, respectively.

In 1989, Ireland* et al*. isolated a tetracyclic alkaloid, diplamine (**117**), from the tunicate *Diplosoma* sp. collected from the Fiji Islands [119]. Diplamine (**117**) was found to be cytotoxic towards L-1210 murine leukemia cells, with an IC_50_ value of 0.02 μg/mL. Two novel alkaloids, isodiplamine (**118**) and lissoclinidine (**119**), along with the previously known diplamine (**117**), were isolated from an ascidian *Lissoclinum notti* collected near Leigh Harbour, Northland, New Zealand (Figure 14). All the compounds (**117**–**119**) were tested for their cytotoxicity against murine leukemia (P-388), human colon tumor (HCT-116), and non-malignant African Green Monkey kidney (BSC-1) cell lines. Diplamine (**117**) was found to be the most active compound among the three and it was observed that movement of the thiomethyl group from C-9 (diplamine) to C-5 (isodiplamine) decreases cytotoxicity against all the cell lines and the same pattern also observed, when the thiomethyl group is cyclized into a benzoxathiole ring (lissoclinidine). These results were also found to be consistent with the proposed mechanism of cytotoxicity of diplamine, which includes DNA intercalation, inhibition of topoisomerase II, and other DNA processing enzymes and bioreductive activation. Lissoclinidine (**119**) was also evaluated against the NCI 60 cell line panel and demonstrated moderate activity and selectivity with panel average values of GI_50_ = 1.0 μM, TGI = 6.9 μM, and LC_50_ = 29 μM [117].

Schmitz* et al*., isolated amphimedine (**120**) as a sparingly soluble yellow pigment from *Amphimedon* sp. [102]. Later, Ireland* et al*., reported the isolation of a new pyridoacridine, neoamphimedine (**121**), along with amphimedine (**120**), from *Xestospongia* sp. from the Philippines and *Xestospongia cf. carbonaria* from Micronesia [106]. Deoxyamphimedine (**122**), along with compounds **120** and **121**, was isolated from two tropical *Xestospongia* sponges [120]. Amphimedine, neoamphimedine, and deoxyamphimedine have the same skeleton, but they differ in biological activities; this is probably due to the differences in their structures. Neoamphimedine inhibits topoisomerase II, while amphimedine is relatively nontoxic at the same dose level [121] and deoxyamphimedine damages DNA independent of topoisomerase enzymes through the generation of reactive oxygen species [122].

**Figure 15:**
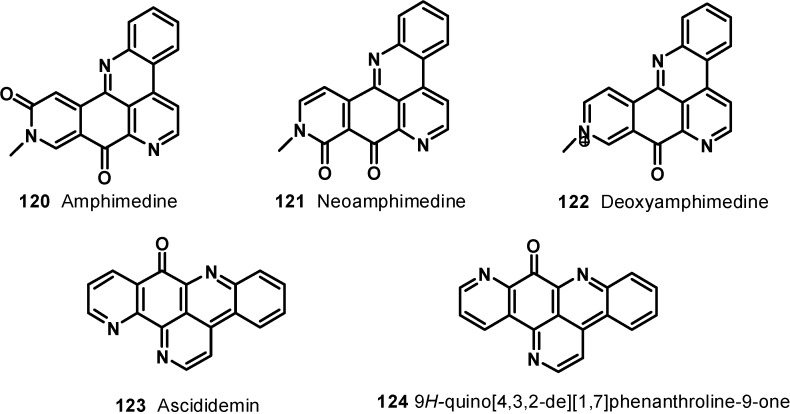
Structures of compounds **120**–**124**.

**Figure 16:**
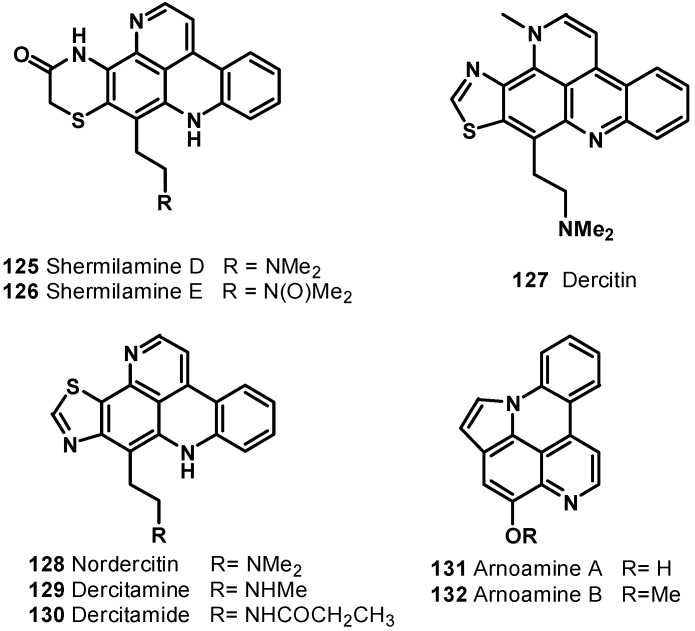
Pentacyclic pyridoacridine alkaloids **125**–**132**.

A pentacyclic alkaloid, ascididemin (**123**), was isolated from brown colored tunicate *Didemnum* sp. collected at Kerama Islands, Okinawa [123]. It was found to be cytotoxic against L-1210 murine leukemia cells* in vitro* with IC_50_ value of 0.39 μg/mL. Delfourne* et al*., synthesized an isomer of ascididemin, named as 9*H*-quino[4,3,2-*de*][1,7]phenanthroline-9-one (**124**) [124]. These compounds were tested *in vitro* at six different concentrations on 12 different human cancer cell lines such as glioblastomas, breast, colon, lung, prostate, and bladder cancers. The compounds showed significant cytotoxic activity; compound **124** was found to be as potent as, or slightly less potent than, the natural ascididemin (**123**) (Figure 15). Further examples of pentacyclic pyridoacridine alkaloids are shermilamine D (**125**) and E (**126**), isolated from the Indian Ocean tunicate *Cystodytes violatinctus* [125,126], dercitin (**127**), isolated from the deep water marine sponge *Dercitus* sp. s [127], nordercitin (**128**), dercitamine (**129**), and dercitamide (**130**), from a *Stelletta* sp. collected in Bahamas [128]. All these compounds were endowed with* in vitro* activity against several tumor cell lines at a micromolar level (Figure 16).

Arnoamines A (**131**) and B (**132**) (Figure 16), isolated from the ascidian *Cystodytes* sp. [129], are the first members of the pentacyclic pyridoacridine alkaloid group, having a pyrrole ring fused with the pyridoacridine ring system. Arnoamine A (**131**) exhibited cytotoxicity against the MCF-7, A-549, and HT-29 cell lines, with GI_50_ values of 0.3, 2.0, and 4.0 μg/mL, respectively, whereas arnoamine B (**132**) showed GI_50_ values of 5.0, 2.0, and 3.0 μg/mL against the MCF-7, A-549, and HT-29 cell lines, respectively.

The methanol extract of the ascidian *Cystodytes dellechiaijei*, collected in Brazil, yielded two novel alkaloids, sebastianine A (**133**) and B (**134**) [130]. Sebastianine A (**133**) was found to be comprised of a pyridoacridine system fused with a pyrrole unit, and sebastianine B (**134**) has a pyridoacridine system fused with a pyrrolidine system condensed with R-hydroxyisovaleric acid. Sebastianine A (**133**) and B (**134**) showed cytotoxic activity against a panel of HCT-116 colon carcinoma cells.

**Figure 17:**
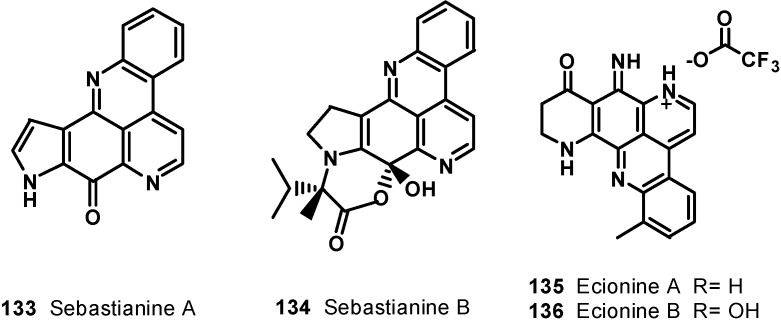
Structures of compounds **133**–**136**.

Ecionines A (**135**) and B (**136**), from the Australian sponge *Ecionemia geodides* [131], contain an imine moiety, which is very rarely found in the pyridoacridine class (Figure 17). Both compounds were tested against a panel of human bladder cancer cell lines (TSU-Pr1, TSU-Pr1-B1, and TSU-Pr1-B2) and the superficial bladder cancer cell line 5637. Compound **136** showed moderate cytotoxicity against all the cell lines, with IC50 values of 6.48 mM (TSU-Pr1), 6.49 mM (TSU-Pr1-B1), 3.55 mM (TSU-Pr1-B2), and 3.66 mM (5637), whereas compound **135** showed cytotoxic effect on 5637 and TSU-Pr1-B2 cells at 10 mM, with cell growth inhibitions of 54% and 51% cells, respectively, but did not have any effect on TSU-Pr1-B1 cells at 10 mM. Compounds **135** and **136** were tested against the human prostate cancer cell line (PC3) and the human neonatal foreskin fibroblast non-cancer cell line (NFF). The results showed that these compounds were approximately 10-fold more selective for prostate cancer PC3 cells, compared with non-cancer NFF cells. This is the first time such selectivity between PC3 and NFF cells by these compounds has been reported [132].

**Figure 18:**
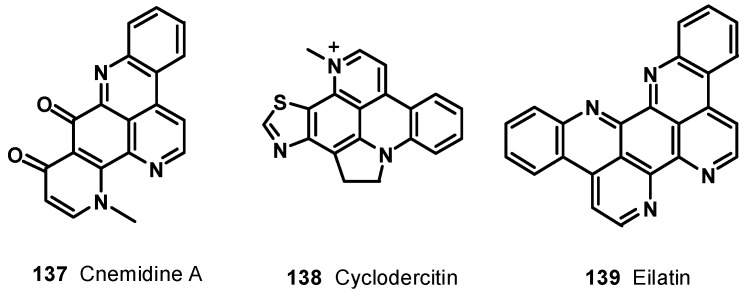
Structures of compounds **137**–**139**.

Chemical investigation of the tunicate *Cnemidocarpa stolonifera* led to the isolation of cnemidine A (**137**) (Figure 18) with a selective cytotoxicity against human prostate cancer cells (PC3, IC_50_ = 1.1 μM) compared with human neonatal foreskin fibroblast non-cancer cells (NFF) [132]. The extracts of a deep violet sponge, *Dercitus* sp., collected in the Bahamas yielded a hexacyclic alkaloid cyclodercitin (**138**). The sixth ring in cyclodercitin (**138**) is formally derived by cyclization of the 2-aminoethyl side chain to the acridine nitrogen, while the pyridine ring is substituted with an *N*-methyl group. Cyclodercitin (**138**) inhibited the proliferation of P-388 murine leukemia cells* in vitro* with an IC_50_ value of 1.9 μM (Figure 18). Eilatin (**139**) isolated from the marine tunicate *Eudistoma* [133] is the only known heptacyclic pyridoacridine alkaloid of marine origin (Figure 18). Eilatin (**139**) was found to exhibit cytotoxic activity against an HCT cell line, with an IC_50_ value of 5.3 μM [134].

## 8. Conclusions

**Figure 19:**
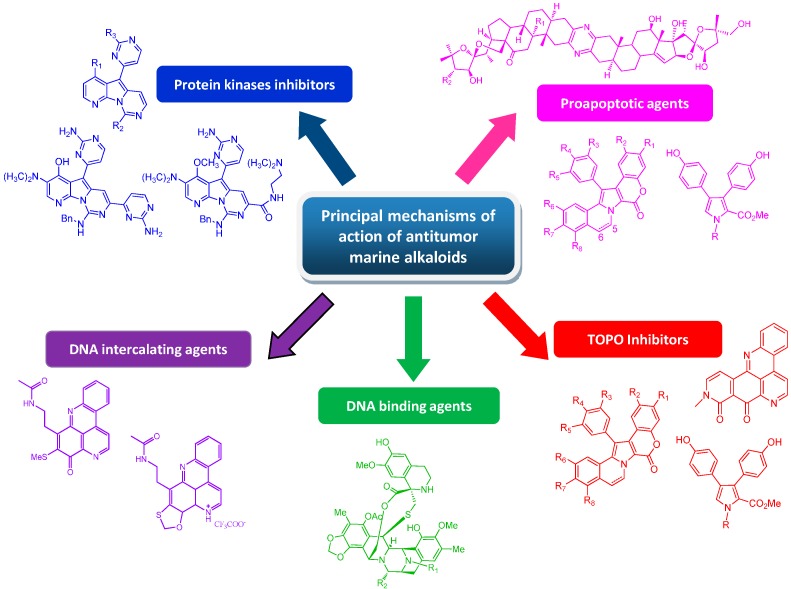
Principal mechanisms of action evidenced for antitumor marine alkaloids.

The undeniable role of marine invertebrate alkaloids as an important source of leads for anticancer drug discovery is now getting its due exposure. The remarkably high chemical diversity of their structures, including indoles, pyrroles, pyrazines, quinolines, pyridoacridines, and other structural families, represents a good resource for new anticancer therapy discovery programs [135,136]. Marine invertebrate alkaloids indeed exhibit varied physiological effects on different targets, ranging from the inhibition of cells’ activity-regulating enzymes (kinases) to DNA-damaging effects (TOPO-inhibition, intercalating agents) and proapoptotic activity (Figure 19). Some of them probably act through novel mechanisms, implying that they could be used to treat drug-resistant cancers. Unfortunately, these reported activities are mostly observed* in vitro*; without* in vivo* data it is difficult to fully appreciate the potential of these drugs. Although the supply issue of marine invertebrate alkaloids still remains, due to their very low availability from natural materials, their potential in medicinal chemistry can now be cleverly exploited. Thanks to current high-yield synthetic methods and recent advances in biotechnology, sufficient material for a broad biological screening, including* in vivo* tests, can be produced. Progress in genetic engineering techniques, like precursor-directed biosynthesis, combinatorial biosynthesis, and mutasynthesis approaches [137,138,139,140], opens up a structural space that is often orthogonal to classical natural product derivatization or total synthesis approaches. Understanding and exploiting the biosynthesis pathways of natural products has an increasing impact on accelerating their discovery and development process. However, the potential utility of marine natural products is not only to make drugs per se but mainly to be used as starting points to develop new drugs and/or therapeutic approaches. As an example, novel chemotypes could be “inspired” by marine natural molecules or even, as in the case of meriolins, “hybrid” frameworks can be designed by combining the chemical features of different compounds acting on the same target. Finally, the recent successful development of the use of monoclonal antibodies (mAbs) directed against targets that are specifically expressed in tumor cells linked to bioactive natural products should be mentioned. The covalent coupling of a targeting monoclonal antibody to a cytotoxic natural product is expected to combine the specificity and tolerance of mAbs with the cell-killing efficiency of natural products in a single molecule, a so-called immunoconjugate or antibody–drug conjugate (ADC) [141]. Therefore, a multidisciplinary approach involving the generation of novel molecular diversity from natural product sources and exploitation of the new methods that have been developed in organic chemistry, biochemistry, molecular biology, and molecular genetics could answer the continuous need for new resources in anticancer drug discovery.

## References

[1] Perry N.B., Ettoouati L., Litaudon M., Blunt J.W., Munro M.H.G., Parkin S., Hope H. (1994). Alkaloids from the antarctic sponge Kirkpatrickia varialosa. Part 1: Variolin B, a new antitumor and antiviral compound. Tetrahedron.

[2] Trimurtulu G., Faulkner D.J., Perry N.B., Ettouati L., Litaudon M., Blunt J.W., Munro M.H.G., Jameson G.B. (1994). Alkaloids from the antarctic sponge Kirkpatrickia varialosa. Part 2: Variolin A and N(3')-methyl tetrahydrovariolin B. Tetrahedron.

[3] Walker S.R., Carter E.J., Huff B.C., Morris J.C. (2009). Variolins and Related Alkaloids. Chem. Rev..

[4] Anderson R.J., Morris J.C. (2001). Total synthesis of variolin B. Tetrahedron Lett..

[5] Anderson R.J., Hill J.B., Morris J.C. (2005). Concise Total Syntheses of Variolin B and Deoxyvariolin B. J. Org. Chem..

[6] Simone M., Erba E., Damia G., Vikhanskaya F., Francesco A.M.D., Riccardi R., Bailly C., Cuevas C., Sousa-Faro J.M.F., D’Incalci M. (2005). Variolin B and its derivate deoxy-variolin B: New marine natural compounds with cyclin-dependent kinase inhibitor activity. Eur. J. Cancer.

[7] Bettayeb K., Tirado O.M., Marionneau-Lambot S., Ferandin Y., Lozach O., Morris J.C., Mateo-Lozano S., Drueckes P., Schächtele C., Kubbutat M.H.G. (2007). Meriolins, a new class of cell death-inducing Kinase inhibitors with enhanced selectivity for cyclin-dependent Kinases. Cancer Res..

[8] Echalier A., Bettayeb K., Ferandin Y., Lozach O., Clement M., Valette A., Liger F., Marquet B., Morris J.C., Endicott J.A. (2008). Meriolins (3-(Pyrimidin-4-yl)-7-azaindoles): Synthesis, Kinase Inhibitory Activity, Cellular Effects, and Structure of a CDK2/Cyclin A/Meriolin Complex. J. Med. Chem..

[9] Anderson R.J., Manzanares I., Morris J.C., Remuinan M. (2002). Variolin Derivatives as Anti-Cancer Agents.

[10] Remuinan M., Gonzalez J., del Pozo C., Francesch A., Cuevas C., Munt S., Manzanares I., Morris J.C., Anderson R. (2003). Variolin Derivatives and Their Use as Antitumour Agents.

[11] Alvarez M., Fernandez Bleda D., Fernandez Puentes J.L. (2002). Preparation of Variolin B Derivatives as Antitumor Agents.

[12] Fresneda P.M., Delgado S., Francesch A., Manzanares I., Cuevas C., Molina P. (2006). Synthesis and Cytotoxic Evaluation of New Derivatives of the Marine Alkaloid Variolin B. J. Med. Chem..

[13] Remuinan M., Gonzalez J., del Pozo C., Francesh A., Cuevas C., Munt S., Manzanares I., Anderson R.J., Morris J.C. (2010). Variolin Derivatives and their Use as Antitumor Agents. U.S. Patent.

[14] Franco L.H., Joffe E.B., Puricelli L., Tatian M., Seldes A.M., Palermo J.A. (1998). Indole alkaloids from the tunicate *Aplidium meridianum*. J. Nat. Prod..

[15] Seldes A.M., Brasco M.F.R., Franco L.H., Palermo J.A. (2007). Identification of two meridianins from the crude extract of the tunicate *Aplidium meridianum* by tandem mass spectrometry. Nat. Prod. Res..

[16] Gompel M., Leost M., de Kier J.E.B., Puricelli L., Franco L.H., Palermo J., Meijer L. (2004). Meridianins, a new family of protein kinase inhibitors isolated from the ascidian *Aplidium meridianum*. Bioorg. Med. Chem. Lett..

[17] Reyes F., Fernandez R., Rodriguez A., Francesch A., Taboada S., Avila C., Cuevas C. (2008). Aplicyanins A-F, new cytotoxic bromoindole derivatives from the marine tunicate *Aplidium cyaneum*. Tetrahedron.

[18] Bartik K., Braekman J.-C., Daloze D., Stoller C., Huysecom J., Vandevyver G., Ottinger R. (1987). Topsentins, new toxic bis-indole alkaloids from the marine sponge *Topsentia genitrix*. Can. J. Chem..

[19] Murray L.M., Lim T.K., Hooper J.N.A., Capon R.J. (1995). Isobromotopsentin: A new bis(indole) alkaloid from a deep-water marine sponge *Spongosorites* sp. Aust. J. Chem..

[20] Tsujii S., Rinehart K.L., Gunasekera S.P., Kashman Y., Cross S.S., Lui M.S., Pomponi S.A., Diaz M.C. (1988). Topsentin, bromotopsentin, and dihydrodeoxybromotopsentin: Antiviral and antitumor bis(indolyl)imidazoles from Caribbean deep-sea sponges of the family *Halichondriidae*. Structural and synthetic studies. J. Org. Chem..

[21] Casapullo A., Bifulco G., Bruno I., Riccio R. (2000). New bisindole alkaloids of the Topsentin and Hamacanthin classes from the Mediterranean marine sponge *Rhaphisia lacazei*. J. Nat. Prod..

[22] Morris S.A., Andersen R.J. (1989). Nitrogenous metabolites from the deep water sponge *Hexadella* sp. Can. J. Chem..

[23] Shin J., Seo Y., Cho K.W., Rho J.-R., Sim C.J. (1999). New Bis(Indole) Alkaloids of the Topsentin class from the sponge *Spongosorites genitrix*. J. Nat. Prod..

[24] Sakemi S., Sun H.H. (1991). Nortopsentins A, B, and C. Cytotoxic and antifungal imidazolediylbis[indoles] from the sponge *Spongosorites ruetzleri*. J. Org. Chem..

[25] Sun H.H., Sakemi S., Gunasekera S., Kashman Y., Lui M., Burres N., Mc Carthy P. (1990). Bisindole Compounds which are Useful Antitumor and Antimicrobial Agents. U.S. Patent.

[26] Mancini I., Guella G., Debitus C., Waikedre J., Pietra F. (1996). From inactive nortopsentin D, a novel bis(indole) alkaloid isolated from the axinellid sponge *Dragmacidon* sp. from deep waters south of new caledonia, to a strongly cytotoxic derivative. Helv. Chim. Acta.

[27] Kawasaki I., Yamashita M., Ohta S. (1996). Total synthesis of nortopsentins A-D, marine alkaloids. Chem. Pharm. Bull..

[28] Jiang B., Gu X.-H. (2000). Syntheses and cytotoxicity evaluation of bis(indolyl)thiazole, bis(indolyl)pyrazinone and bis(indolyl)pyrazine: Analogues of cytotoxic marine bis(indole) alkaloid. Bioorg. Med. Chem..

[29] Moser B.R. (2008). Review of cytotoxic cephalostatins and ritterazines: Isolation and synthesis. J. Nat. Prod..

[30] Lee S., LaCour T.G., Fuchs P.L. (2009). Chemistry of trisdecacyclic pyrazine antineoplastics: The cephalostatins and ritterazines. Chem. Rev..

[31] Menna M., Fattorusso E., Imperatore C. (2011). Alkaloids from Marine Ascidians. Molecules.

[32] Komiya T., Fusetani N., Matsunaga S., Kubo A., Kaye F.J., Kelley M.J., Tamura K., Yoshida M., Fukuoka M., Nakagawa K. (2003). Ritterazine B, a new cytotoxic natural compound, induces apoptosis in cancer cells. Cancer Chemother. Pharmacol..

[33] Ganesan A. (1996). The dimeric steroid-pyrazine marine alkaloids: Challenges for isolation, synthesis, and biological studies. Angew. Chem. Int. Ed. Engl..

[34] López-Antón N., Rudy A., Barth N., Schmitz L.M., Pettit G.R., Schulze-Osthoff K., Dirsch V.M., Vollmar A.M. (2006). The marine product cephalostatin 1 activates and ER stress-specific and apoptosome- independent apoptotic signaling pathway. J. Biol. Chem..

[35] Rudy A., López-Antón N., Dirsch V.M., Vollmar A.M. (2008). The Cephalostatin Way of Apoptosis. J. Nat. Prod..

[36] Rudy A., López-Antón N., Barth N., Pettit G.R., Dirsch V.M., Schulze-Osthoff K., Rehm M., Prehn J.H.M., Vogler M., Fulda S. (2008). Role of Smac in cephalostatin-induced cell death. Cell Death Differ..

[37] Cironi P., Albericio F., Alvarez M. (2004). Lamellarins: Isolation, activity and synthesis. Prog. Heterocycl. Chem..

[38] Bailly C. (2004). Lamellarins, from A to Z: A family of anticancer marine pyrrole alkaloids. Curr. Med. Chem. Anti-Cancer Agents.

[39] Fan H., Peng J., Hamann M.T., Hu J.F. (2008). Lamellarins and related pyrrole-derived alkaloids from marine organisms. Chem. Rev..

[40] Kluza J., Marchetti P., Bailly C. (2008). Lamellarin alkaloids: Structure and pharmacological properties. Modern Alkaloids: Structure, Isolation, Synthesis and Biology.

[41] Pla D., Albericio F., Alvarez M. (2011). Progress on lamellarins. Med. Chem. Commun..

[42] Andersen R.J., Faulkner D.J., He C.H., van Duyne G.D., Clardy J. (1985). Metabolites of the marine prosobranch mollusk *Lamellaria* sp. J. Am. Chem. Soc..

[43] Lindquist N., Fenical W., van Duyne G.D., Clardy J. (1988). New alkaloids of the Lamellarin class from the marine ascidian *Didemnum chartaceum*. J. Org. Chem..

[44] Carroll A.R., Bowden B.F., Coll J.C. (1993). Studies of Australian ascidians. I. Six new lamellarin class alkaloids from a colonial ascidian, *Didemnum* sp. Aust. J. Chem..

[45] Davis R.A., Carroll A.R., Pierens G.K., Quinn R.J. (1999). New lamellarin alkaloids from the Australian ascidian *Didemnum chartaceum*. J. Nat. Prod..

[46] Reddy M.V.R., Rao M.R., Rhodes D., Hansen M.S.T., Rubins K., Bushman F.D., Venkateswarlu Y., Faulkner D.J. (1999). Lamellarin α-20 sulfate, an inhibitor of HIV-1 integrase active against HIV-1 virus in cell culture. J. Med. Chem..

[47] Urban S., Capon R.J. (1996). Lamellarin-S: A new aromatic metabolite from an Australian Tunicate, *Didemnum* sp. Aust. J. Chem..

[48] Reddy S.M., Srinivasulu M., Satyanarayana N., Kondapi A.K., Venkateswarlu Y. (2005). New potent cytotoxic lamellarin alkaloids from Indian ascidian *Didemnum obscurum*. Tetrahedron.

[49] Urban S., Butler M.S., Capon R.J. (1994). Lamellarins O and P: New aromatic metabolites from the Australian marine sponge *Dendrilla cactos*. Aust. J. Chem..

[50] Urban S., Hobbs L., Hooper J.N.A., Capon R.J. (1995). Lamellarins Q and R: New aromatic metabolites from an Australian marine Sponge *Dendrilla cactos*. Aust. J. Chem..

[51] Quesada A.R., Garcia Gravalos M.D., Fernandez Puentes J.L. (1996). Polyaromatic alkaloids from marine invertebrates as cytotoxic compounds and inhibitors of multidrug resistance caused by Pglycoprotein. Br. J. Cancer.

[52] Urban S., Hickford S.J.H., Blunt J.W., Munro M.H.G. (2000). Bioactive marine alkaloids. Curr. Org. Chem..

[53] Facompre M., Tardy C., Bal-Mahieu C., Colson P., Perez C., Manzanares I., Cuevas C., Bailly C. (2003). Lamellarin D: A novel potent inhibitor of topoisomerase I. Cancer Res..

[54] Tardy C., Facompre M., Laine W., Baldeyrou B., Garcia-Gravalos D., Francesch A., Mateo C., Pastor A., Jimenez J.A., Manzanares I. (2004). Topoisomerase I-mediated DNA cleavage as a guide to the development of antitumor agents derived from the marine alkaloid lamellarin D: Trimethylester derivatives incorporating amino acid residues. Bioorg. Med. Chem..

[55] Marco E., Laine W., Tardy C., Lansiaux A., Iwao M., Ishibashi F., Bailly C., Gago F. (2005). Molecular determinants of topoisomerase I poisoning by lamellarins: Comparison with camptothecin and structure-activity relationships. J. Med. Chem..

[56] Vanhuyse M., Kluza J., Tardy C., Otero G., Cuevas C., Bailly C., Lansiaux A. (2005). Lamellarin D: A novel pro-apoptotic agent from marine origin insensitive to P-glycoprotein-mediated drug efflux. Cancer Lett..

[57] Kluza J., Gallego M.A., Loyens A., Beauvillain J.C., Sousa- Faro J.M., Cuevas C., Marchetti P., Bailly C. (2006). Cancer cell mitochondria are direct proapoptotic targets for the marine antitumor drug lamellarin D. Cancer Res..

[58] Gallego M.A., Ballot C., Kluza J., Hajji N., Martoriati A., Castéra L., Cuevas C., Formstecher P., Joseph B., Kroemer G. (2008). Overcoming chemoresistance of non-small cell lung carcinoma through restoration of an AIF-dependent apoptotic pathway. Oncogene.

[59] Pla D., Marchal A., Olsen C.A., Francesch A., Cuevas C., Albericio F., Alvarez M. (2006). Synthesis and Structure Activity Relationship Study of Potent Cytotoxic Analogues of the Marine Alkaloid Lamellarin D. J. Med. Chem..

[60] Chittchang M., Batsomboon P., Ruchirawat S., Ploypradith P. (2009). Cytotoxicities and structure—Activity relationships of natural and unnatural lamellarins toward cancer cell Lines. Chem. Med. Chem..

[61] Khiati S., Seol Y., Agama K., Dalla Rosa I., Agrawal S., Fesen K., Zhang H., Neuman K.C., Pommie Y. (2014). Poisoning of mitochondrial topoisomerase I by lamellarin D. Mol. Pharmacol..

[62] Chittchang M., Gleeson M.P., Ploypradith P., Ruchirawat S. (2010). Assessing the drug-likeness of lamellarins, a marine-derived natural product class with diverse oncological activities. Eur. J. Med. Chem..

[63] Le V.H., Inai M., Williams R.M., Kan T. (2014). Ecteinascidins. A review of the chemistry, biology and clinical utility of potent tetrahydroisoquinoline antitumor antibiotics. Nat. Prod. Rep..

[64] Garcia-Carbonero R., Supko J.G., Maki R.G., Manola J., Ryan D.P., Harmon D., Puchalski T.A., Goss G., Seiden M.V., Waxman A. (2005). Ecteinascidin-743 (ET-743) for chemotherapy-naive patients with advanced soft tissue sarcomas: Multicenter phase II and pharmacokinetic study. J. Clin. Oncol..

[65] Cuevas C., Francesch A. (2009). Development of Yondelis^®^ (trabectedin, ET-743). A semisynthetic process solves the supply problem. Nat. Prod. Rep..

[66] Krasner C.N., McMeekin D.S., Chan S., Braly P.S., Renshaw F.G., Kaye S., Provencher D.M., Campos S., Gore M.E. (2007). A Phase II study of trabectedin single agent in patients with recurrent ovarian cancer previously treated with platinum-based regimens. Br. J. Cancer.

[67] Rinehart K.L., Holt T.G., Fregeau N.L., Stroh J.G., Keifer P.A., Sun F., Li L.H., Martin D.G. (1990). Ecteinascidins 729, 743, 745, 759A. 759B, and 770: Potent antitumor agents from the Caribbean tunicate *Ecteinascidia turbinata*. J. Org. Chem..

[68] Wright A.E., Forleo D.A., Gunawardana G.P., Gunasekera S.P., Koehn F.E., McConnell O.J. (1990). Antitumor tetrahydroisoquinoline alkaloids from the colonial ascidian *Ecteinascidia turbinata*. J. Org. Chem..

[69] Guan Y., Sakai R., Rinehart K.L., Wang A.H.J. (1993). Molecular and crystal structures of ecteinascidins: Potent antitumor compounds from the Caribbean tunicate *Ecteinascidia turbinata*. J. Biomol. Struct. Dyn..

[70] Sakai R., Rinehart K.L., Guan Y., Wang A.H.J. (1992). Additional antitumor ecteinascidins from a Caribbean tunicate: Crystal structures and activities* in vivo*. Proc. Natl. Acad. Sci. USA.

[71] Rinehart K.L., Sakai R. (2004). Isolation, Structure Elucidation, and Bioactivities of Novel Ecteinascidins from *Ecteinascidia turbinata*. U.S. Patent.

[72] Suwanborirux K., Charupant K., Amnuoypol S., Pummangura S., Kubo A., Saito N. (2002). Ecteinascidins 770 and 786 from the Thai Tunicate *Ecteinascidia thurstoni*. J. Nat. Prod..

[73] Sakai R., Jares-Erijman E.A., Manzanares I., Elipe M.V.S., Rinehart K.L. (1996). Ecteinascidins: Putative biosynthetic precursors and absolute stereochemistry. J. Am. Chem. Soc..

[74] Scott J.D., Williams R.M. (2002). Chemistry and Biology of the Tetrahydroisoquinoline Antitumor Antibiotics. Chem. Rev..

[75] Soares D.G., Larsen A.K., Escargueil A.E. (2012). The DNA damage response to monofunctional anticancer DNA binders. Drug Discov. Today: Dis. Models.

[76] Aune G.J., Furuta T., Pommier Y. (2002). Ecteinascidin 743: A novel anticancer drug with a unique mechanism of action. Anticancer Drugs.

[77] Zewail-Foote M., Hurley L.H. (1999). Ecteinascidin 743: A minor groove alkylator that bends DNA toward the major groove. J. Med. Chem..

[78] Takebayashi Y., Pourquier P., Zimonjic D.B., Nakayama K., Emmert S., Ueda T., Urasaki Y., Kanzaki A., Akiyama S.-I., Popescu N. (2001). Antiproliferative activity of ecteinascidin 743 is dependent upon transcription- coupled nucleotide-excision repair. Nat. Med..

[79] D’Incalci M., Erba E., Damia G., Galliera E., Carrassa L., Marchini S., Mantovani R., Tognon G., Fruscio R., Jimeno J. (2002). Unique features of the mode of action of ET-743. Oncologist.

[80] Fayette J., Coquard I.R., Alberti L., Boyle H., Meeus P., Decouvelaere A.V., Thiesse P., Sunyach M.P., Ranchere D., Blay J.Y. (2006). ET-743: A novel agent with activity in soft-tissue sarcomas. Curr. Opin. Oncol..

[81] Christinat A., Leyvraz S. (2009). Role of trabectedin in the treatment of soft tissue sarcoma. Onco Targets Ther..

[82] Gajdos C., Elias A. (2011). Trabectedin: Safety and efficacy in the treatment of advanced sarcoma. Clin. Med. Insight Oncol..

[83] Pharmamar. http://www.yondelis.com/yondelis.aspx.

[84] Carballo J.L., Naranho S., Kukurtzu B., de La Calle F., Hernandez-Zanuy A. (2000). Production of *Ecteinascidia turbinate* (Ascidiacea: Perophoridae) for obtaining anticancer compounds. J. World Aquac. Soc..

[85] Corey E.J., Gin D.Y., Kania R.S. (1996). Enantioselective total synthesis of Ecteinascidin 743. J. Am. Chem. Soc..

[86] Martinez E., Corey E.J. (2000). A New, More Efficient, and Effective Process for the Synthesis of a Key Pentacyclic Intermediate for Production of Ecteinascidin and Phthalascidin Antitumor Agents. Org. Lett..

[87] Endo A., Yanagisawa A., Abe M., Tohma S., Kan T., Fukuyama T. (2002). Total Synthesis of Ecteinascidin 743. J. Am. Chem. Soc..

[88] Chen J., Chen X., Bois-Choussy M., Zhu J. (2006). Total Synthesis of Ecteinascidin 743. J. Am. Chem. Soc..

[89] Zheng S., Chan C., Furuuchi T., Wright B.J.D., Zhou B., Gio Z.J., Danishefsky S.J. (2006). Stereospecific Formal Total Synthesis of Ecteinascidin 743. Angew. Chem. Int. Ed..

[90] Fishlock D., Williams R.M. (2008). Synthetic Studies on Et-743. Assembly of the Pentacyclic core and a formal total synthesis. J. Org. Chem..

[91] Avendaño C., de la Cuesta E. (2010). Recent synthetic approaches to 6,15-inoisoquino[3,2-b]3-benzazocine compounds. Chem. Eur. J..

[92] Cuevas C., Perez M., Martin M.J., Chicharro J.L., Fernandez-Rivas C., Flores M., Francesch A., Gallego P., Zarzuelo M., de la Calle F. (2000). Synthesis of ecteinascidin ET-743 and phthalascidin Pt-650 from cyanosafracin B. Org Lett..

[93] Menchaca R., Martínez V., Rodríguez A., Rodríguez N., Flores M., Gallego P., Manzanares I., Cuevas C. (2003). Synthesis of natural Ecteinascidins (ET-729, ET-745, ET-759B, ET-736, ET-637, ET-594) from Cyanosafracin B. J. Org. Chem..

[94] Martinez E.J., Owa T., Schreiber S.L., Corey E.J. (1999). Phtalascidin, a synthetic antitumor agent with potency and mode of action comparable to ecteinascidin 743. Proc. Natl. Acad. Sci. USA.

[95] Garcia-Nieto R., Manzanares I., Cuevas C., Gago F. (2000). Bending of DNA upon binding of ecteinascidin 743 and phthalascidin 650 studied by unrestrained molecular dynamics simulations. J. Am. Chem. Soc..

[96] Martinez E.J., Corey E.J., Owa T. (2001). Antitumor activity- and gene expression-based profiling of ecteinascidin Et743 and phtalascidin Pt 650. Chem. Biol..

[97] Leal J.F.M., Martínez-Díez M., García-Hernández V., Moneo V., Domingo A, Bueren-Calabuig J.A., Negri A., Gago F., Guillén-Navarro M.J., Avilés P. (2010). PM01183, a new DNA minor groove covalent binder with potent* in vitro* and* in vivo* anti-tumour activity. Br. J. Pharmacol..

[98] Soares D.G., Machado M.S., Rocca C.J., Poindessous V., Ouaret D., Sarasin A., Galmarini C.M., Henriques G.A.P., Escargueil A.E., Larsen A.K. (2011). Trabectedin and Its C Subunit Modified Analogue PM01183 Attenuate Nucleotide Excision Repair and Show Activity toward Platinum-Resistant Cells. Mol. Cancer. Ther..

[99] Pharmamar. http://www.pharmamar.com/busqueda.aspx.

[100] Delfourne E., Bastide J. (2003). Marine pyridoacridine alkaloids and synthetic analogues as antitumor agents. Med. Res. Rev..

[101] Lederer E., Teissier G., Huttrer C. (1940). The isolation and chemical composition of calliactine, pigment of the sea anemone “*Sagartia parasitica*” (Calliactis effoeta). Bull. Soc. Chim. Fr..

[102] Schmitz F.J., Agarwal S.K., Gunasekera S.P., Schmidt P.G., Shoolery J.N. (1983). Amphimedine, new aromatic alkaloid from a pacific sponge, *Amphimedon* sp. Carbon connectivity determination from natural abundance carbon-13-carbon-13 coupling constants. J. Am. Chem. Soc..

[103] Molinski T.F. (1993). Marine pyridoacridine alkaloids: Structure, synthesis, and biological chemistry. Chem. Rev..

[104] Bontemps N., Gattacceca F., Long C., Thomas O.P., Banaigs B. (2013). Additional Cytotoxic Pyridoacridine Alkaloids from the Ascidian *Cystodytes violatinctus* and Biogenetic Considerations. J. Nat. Prod..

[105] Marshall K.M., Barrows L.R. (2004). Biological activities of pyridoacridines. Nat. Prod. Rep..

[106] De Guzman F.S., Carte B., Troupe N., Faulkner D.J., Harper M.K., Concepcion G.P., Mangalindan G.C., Matsumoto S.S., Barrows L.R., Ireland C.M. (1999). Neoamphimedine: A new pyridoacridine topoisomerase II inhibitor which catenates DNA. J. Org. Chem..

[107] Feng Y.R., Davis A., Sykes M.L., Avery V.M., Carroll A.R., Camp D., Quinn R.J. (2010). Antitrypanosomal pyridoacridine alkaloids from the Australian ascidian *Polysyncraton echinatum*. Tetrahedron Lett..

[108] Fuente J.A.D.L., Martın M.J., Blanco M.D.M., Alfonso E.P., Avendano C., Menendez J.C. (2001). A C-Ring regioisomer of the marine alkaloid meridine exhibits selective* in vitro* cytotoxicity for solid tumours. Bioorg. Med. Chem..

[109] Schmitz F.J., Deguzman F.S., Hossain M.B., Helm D.V.D. (1991). Cytotoxic aromatic alkaloids from the ascidian *Amphicarpa meridiana* and *Leptoclinides* sp.: Meridine and 11-hydroxyascididemin. J. Org. Chem..

[110] McCarthy P.J., Pitts T.P., Gunawardana G.P., Borges M.K., Pomponi S.A. (1992). Antifungal activity of meridine, a natural product from the marine sponge *Corticium* sp. J. Nat. Prod..

[111] Carroll A.R., Ngo A., Quinn R.J., Redburn J., Hooper J.N.A. (2005). Petrosamine B, an inhibitor of the *Helicobacter pylori* enzyme aspartyl semialdehyde dehydrogenase from the Australian sponge *Oceanapia* sp. J. Nat. Prod..

[112] Skyler D., Heathcock C.H. (2002). The Pyridoacridine Family Tree: A Useful Scheme for Designing Synthesis and Predicting Undiscovered Natural Products. J. Nat. Prod..

[113] Antunes E.M., Copp B.R., Coleman M.T.D., Samaai T. (2005). Pyrroloiminoquinone and related metabolites from marine sponges. Nat. Prod. Rep..

[114] Kobayashi J., Cheng J.F., Walchli M.R., Nakamura H., Hirata Y., Sasaki T., Ohizumi Y. (1988). Cystodytins A, B, and C, novel tetracyclic aromatic alkaloids with potent antineoplastic activity from the Okinawan tunicate *Cystodytes dellechiajei*. J. Org. Chem..

[115] Kobayashi J., Tsuda M., Tanabe M., Ishibashi M. (1991). Cystodytins D-I, new cytotoxic tetracyclic aromatic alkaloids from the Okinawan marine tunicate *Cystodytes dellechiajei*. J. Nat. Prod..

[116] McDonald L.A., Eldredge G.S., Barrows L.R., Ireland C.M. (1994). Inhibition of topoisomerase II catalytic activity by pyridoacridine alkaloids from a *Cystodytes* sp. ascidian: A mechanism for the apparent intercalator-induced inhibition of topoisomerase II. J. Med. Chem..

[117] Appleton D.R., Pearce A.N., Lambert G., Babcockc R.C., Copp B.R. (2002). Isodiplamine, cystodytin K and lissoclinidine: Novel bioactive alkaloids from the New Zealand ascidian *Lissoclinum nott*. Tetrahedron.

[118] Molinski T.F., Ireland C.M. (1989). Varamines A and B, new cytotoxic thioalkaloids from *Lissoclinum vareau*. J. Org. Chem..

[119] Charyulu G.A., McKee T.C., Ireland C.M. (1989). Diplamine, a cytotoxic polyaromatic alkaloid from the tunicate *Diplosoma* sp. Tetrahedron Lett..

[120] Tasdemir D., Marshall K.M., Mangalindan G.C., Concepcion G.P., Barrows L.R., Harper M.K., Ireland C.M. (2001). Deoxyamphimedine, a new pyridoacridine alkaloid from two tropical *Xestospongia* sponges. J. Org. Chem..

[121] Marshall K.M., Matsumoto S.S., Holden J.A., Concepcion G.P., Tasdemir D., Ireland C.M., Barrows L.R. (2003). The anti-neoplastic and novel topoisomerase II-mediated cytotoxicity of neoamphimedine, a marine pyridoacridine. Biochem. Pharmacol..

[122] Marshall K.M., Andjelic C.D., Tasdemir D., Concepción G.P., Ireland C.M., Barrows L.R. (2009). Deoxyamphimedine, a pyridoacridine alkaloid, damages DNA via the production of reactive oxygen species. Mar. Drugs.

[123] Kobayashi J., Cheng J.F., Nakamura H., Ohizumi Y., Hirata Y., Sasaki T., Ohta T., Nozoe S. (1988). Ascididemin, a novel pentacyclic aromatic alkaloid with potent antileukemic activity from the Okinawan tunicate *Didemnum* sp. Tetrahedron Lett..

[124] Delfourne E., Kiss R., le Corre L., Merza J., Bastide J., Frydman A., Darro F. (2003). Synthesis and* in vitro* antitumor activity of an isomer of the marine pyridoacridine alkaloid ascididemin and related compounds. Bioorg. Med. Chem..

[125] Koren-Goldshlager G., Aknin M., Gaydou E.M., Kashman Y. (1998). Three New Alkaloids from the Marine Tunicate *Cystodytes violatinctus*. J. Org. Chem..

[126] Kashman Y., Koren-Goldshlager G., Aknin M., Garcia Gravalos D. (1999). Isolation and Characterization of a Cytotoxic Pyridoacridine Alkaloid, Shermilamine D, from *Cystodytes violatinctus*, and its Antitumor Activity.

[127] Gunawardana G.P., Kohmoto S., Gunasekera S.P., McConnell O.J., Koehn F.E. (1988). Dercitine, a new biologically active acridine alkaloid from a deep water marine sponge, *Dercitus* sp. J. Am. Chem. Soc..

[128] Gunawardana G.P., Kohmoto S., Burres N.S. (1989). New cytotoxic acridine alkaloids from two deep water marine sponges of the family *Pachastrellidae*. Tetrahedron Lett..

[129] Plubrukarn A., Davidson B.S. (1998). Arnoamines A and B, new cytotoxic pentacyclic pyridoacridine alkaloids from the ascidian *Cystodytes* sp. J. Org. Chem..

[130] Torres Y.R., Bugni T.S., Berlinck R.G.S., Ireland C.M., Magalhaes A., Ferreira A.G., Moreira da Rocha R. (2002). Sebastianines A and B, novel biologically active pyridoacridine alkaloids from the Brazilian ascidian *Cystodytes dellechiajei*. J. Org. Chem..

[131] Barnes E.C., Akmarina S.N., Elizabeth D.W., Hooper J.N.A., Davis R.A. (2010). Ecionines A and B, two new cytotoxic pyridoacridine alkaloids from the Australian marine sponge, *Ecionemia geodides*. Tetrahedron.

[132] Tran T.D., Pham N.B., Quinn R.J. (2014). Structure determination of pentacyclic pyridoacridine alkaloids from the Australian marine organisms *Ancorina geodides* and *Cnemidocarpa stolonifera*. Eur. J. Org. Chem..

[133] Rudi A., Benayahu Y., Goldberg I., Kashman Y. (1988). Eilatin, a novel alkaloid from the marine tunicate *Eudistoma* sp. Tetrahedron Lett..

[134] Rudi A., Kashman Y. (1989). Six new alkaloids from the purple Red Sea tunicate *Eudistoma* sp. J. Org. Chem..

[135] Newman D.J., Cragg G.M. (2012). Natural products as sources of new drugs over the 30 years from 1981 to 2010. J. Nat. Prod..

[136] Koehn F.E., Carter G.T. (2005). The evolving role of natural products in drug discovery. Nat. Rev. Drug Discov..

[137] Witting K., Süssmuth R.D. (2011). Discovery of antibacterials and other bioactive compounds from microorganisms-evaluating methodologies for discovery and generation of non-ribosomal peptide antibiotics. Curr. Drug Targets.

[138] Kennedy J. (2008). Mutasynthesis, chemobiosynthesis, and back to semi-synthesis. Combining synthetic chemistry and biosynthetic engineering for diversifying natural products. Nat. Prod. Rep..

[139] Kirschning A., Hahn F. (2012). Merging chemical synthesis and biosynthesis: A new chapter in the total synthesis of natural products and natural product libraries. Angew. Chem. Int. Ed..

[140] Kirschning A., Taft F., Knobloch T. (2007). Total synthesis approaches to natural product derivatives based on the combination of chemical synthesis and metabolic engineering. Org. Biomol. Chem..

[141] Bauer A., Bronstrup M. (2014). Industrial natural product chemistry for drug discovery and development. Nat. Prod. Rep..

